# Bispecific mAb^2^ Antibodies Targeting CD59 Enhance the Complement-Dependent Cytotoxicity Mediated by Rituximab

**DOI:** 10.3390/ijms23095208

**Published:** 2022-05-06

**Authors:** Katharina Stadlbauer, Peter Andorfer, Gerhard Stadlmayr, Florian Rüker, Gordana Wozniak-Knopp

**Affiliations:** Christian Doppler Laboratory for Innovative Immunotherapeutics, Institute of Molecular Biotechnology, Department of Biotechnology, University of Natural Resources and Life Sciences, Vienna (BOKU), Muthgasse 18, 1190 Vienna, Austria; katharina.stadlbauer@boku.ac.at (K.S.); peterandorfer@gmx.at (P.A.); gerhard.stadlmayr@boku.ac.at (G.S.); florian.rueker@boku.ac.at (F.R.)

**Keywords:** bispecific antibody, CD20, CD59, complement-dependent cytotoxicity, complement regulatory protein, Fcab, mAb^2^, rituximab

## Abstract

Inhibition of complement activation via the overexpression of complement-regulatory proteins (CRPs), most notably CD46, CD55 and CD59, is an efficient mechanism of disguise of cancer cells from a host immune system. This phenomenon extends to counteract the potency of therapeutic antibodies that could lyse target cells by eliciting complement cascade. The manifold functions and ubiquitous expression of CRPs preclude their systemic specific inhibition. We selected CD59-specific Fc fragments with a novel antigen binding site (Fcabs) from yeast display libraries using recombinant antigens expressed in bacterial or mammalian cells. To produce a bispecific antibody, we endowed rituximab, a clinically applied anti-CD20 antibody, used for therapy of various lymphoid malignancies, with an anti-CD59 Fcab. This bispecific antibody was able to induce more potent complement-dependent cytotoxicity for CD20 and CD59 expressing Raji cell line measured with lactate dehydrogenase-release assay, but had no effect on the cells with lower levels of the primary CD20 antigen or CD20-negative cells. Such molecules are promising candidates for future therapeutic development as they elicit a higher specific cytotoxicity at a lower concentration and hence cause a lower exhaustion of complement components.

## 1. Introduction

Activation of the complement system is a highly conserved mechanism of innate immunity, intended for immediate recognition and efficient elimination of foreign intruders [1]. Detection of “danger” signals, molecular signatures present on the surface of intruders, but also infected and damaged cells, is followed by triggering proteolytic events in three cascading pathways, classical, alternative and mannose-binding lectin pathway, which all lead to assembly to multiprotein complexes, C3 and C5 convertases, central to the complement activity [2,3]. Cleaved-off fragments of these bioactive complexes opsonize target surfaces and induce phagocytic and immunomodulatory events, while the terminal lytic pathway leads to the destruction of complement-targeted cells through the formation of membrane attack complex (MAC). Fragment C5b, resulting from the activity of C5 convertase, initiates the MAC assembly with the collaboration of C6 and C7 [4] and eventually engages C8 with C8α subunit as the first component to penetrate the lipid bilayer of the cell membrane [5]. Subsequent association of the oligomeric assembly with up to 18 C9 subunits leads to the formation of the final pore [5].

In this way, MAC can directly kill Gram-negative bacteria [6,7], parasites [8], but it can also assemble on the surface of host cells. Assembled MAC on host cells can result either in direct lysis or apoptosis, depending on the amount of the available MAC components [9]. For non-nucleated cells, such as erythrocytes, a “single-hit” is sufficient to trigger lysis [10]. This clearly demonstrates the requirement of evolution of MAC-counteracting components, allowing fine tuning of its activity and prevention of excessive cellular damage, including killing of bystander cells. Formation of the final complex can be inhibited by the interaction with glycosylphosphatidylinositol (GPI)-linked protein CD59, which can bind either to C8 or C9. Interestingly, CD59 cannot bind to soluble C8 or C9, and it was even postulated that the relevant interactions occur only upon the insertion of these proteins into the membrane [11]. The binding site of CD59 has been mapped onto the amino acid residues of C8α [12] and C9 [13], located in the transmembrane helices. The active site of CD59 has been determined by mutagenesis studies to be facing away from the membrane and include non-contiguous residues, forming a hydrophobic groove [14,15], however also mutations of sterically remote amino acids on the opposite side of the molecule can critically influence its complement inhibitory competence [16]. In the absence of co-crystal of either of the two complement components with CD59, the structural data on the interaction of intermedilysin and CD59 hint towards the possibility that in the final complex of any of the three interaction partners the beta hairpin of CD59 is extended towards the core beta-sheets of the complement constituents, once the surrounding strands had set it sterically available [17]. 

Differences in the expression level of CD59 have a strong influence on complement activity: genetic deficiencies in GPI expression prevent cellular trafficking and surface expression of CD59, which results in paroxysmal nocturnal hemoglobinuria [18]. On the other hand, increased abundancy of CD59 on lymphoid cells can render them refractory to the complement triggering activity of well-established therapeutic antibodies, such as rituximab [19,20]. Even enzymatic removal of CD59 via cleavage of the GPI anchor was sufficient to render a Her2-positive/CD59-positive cell line sensitive to complement-dependent cytotoxic activity of the anti-Her2 antibody trastuzumab [21]. Cis-platin therapy-induced decline of membrane CRPs (mCRPs) CD55 and CD59 levels on the surface of non-small cell lung cancer cells enabled trastuzumab-mediated complement cytotoxicity [22].

The strategy to enhance the potency of complement activation with the blockade of CD59 activity has already proven successful in in vitro and in vivo mouse models: via the connection of an anti-CD59 blocking single-chain Fv fragment with a CD20-targeting antibody into a single entity, used together with another mCRP (CD55) blocking/CD20 specific antibody, significantly higher numbers of cells were killed in cell lines or cells isolated from patients with chronic lymphocytic leukemia, and the treatment with these bispecific antibodies prevented xenograft development in a severe combined immunodeficiency (SCID) mouse model of human lymphoma [23]. Besides underlining the importance of neutralizing the mCRPs activity, the use of bispecific antibodies appears advantageous in this setting as they selectively address cancer cells and avoid the on-target, off-tumor attacks due to widely distributed CD59 expression.

Among many bispecific formats of antibodies available today, the immunoglobulin-like architectures are favored due to a higher degree of predictability of pharmacokinetic properties and a lower level of proteolytic degradation as observed for example in fusion formats [24]. To address the specific task of enhancing the complement-mediated cytotoxicity of a CD20-targeting antibody, such as rituximab, we applied a symmetric IgG-like bispecific antibody of mAb^2^ format, which has already been accepted into the stage of clinical testing [25,26,27,28]. The interaction with a second antigen, in our case CD59, is mediated through a novel binding site in the C-terminal loops of the CH3 domains in its Fc fragment, an antigen-binding Fc fragment (Fcab) [29]. Relevant binders are selected through affinity screening of a library of Fc mutants, displayed either on phage particles or yeast cells, and the antigen-reactive Fc variants can be incorporated into an IgG-scaffold, chosen at will. The beneficial properties of yeast system, featuring the multivalent display of Fc, which enables isolation of low affinity binders, and the option of efficient affinity maturation via recurrent rounds of directed evolution [30,31], guided the choice of methodology for isolation of an anti-CD59 Fcab, optimally suited to selectively enhance the activity of CDC-triggering anti-CD20 antibodies.

## 2. Results

**Figure 1:**
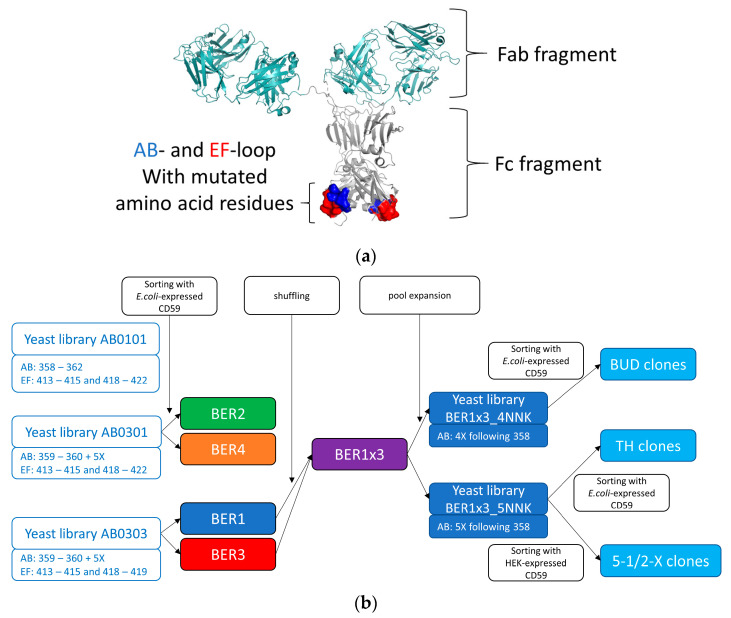
mAb^2^ bispecific molecules contain antigen binding Fc fragments (Fcabs), which can be derived from yeast Fcab libraries: (**a**) Model of a mAb^2^ antibody: Fab fragment in light teal, Fc in gray, and modified residues in the Fc-CH3 domain highlighted in blue (AB loop) and red (EF loop). The Figure was prepared using PyMOL version 2.5 (Schrödinger LLC.) based on PDB:1HZH; (**b**) Scheme of selection and pool expansion of CD59-reactive Fcabs (EU numbering scheme for antibody amino acids is used).

Three Fcab libraries with different designs, AB01EF01, AB03EF01 and AB03EF03 (Figure 1a,b) were used for the selection of CD59-binding clones. The final sizes of the libraries were determined to be 5.37, 3.84 and 5.0 × 10^8^ independent members. All libraries were shown to contain a high percentage of yeast cells binding to structural marker ligands, protein A, conformation dependent anti-CH2 antibody and soluble CD64 even at the stress induction temperature of 37 °C (Supplementary Figure S1).

**Figure 2:**
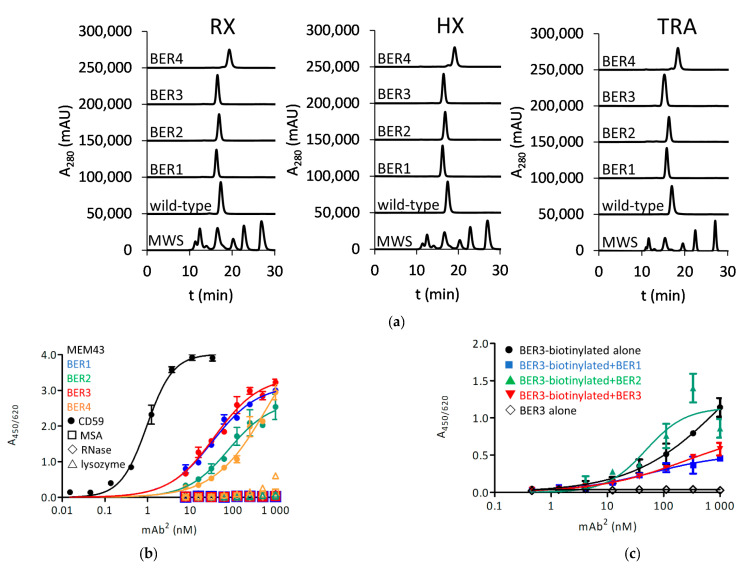

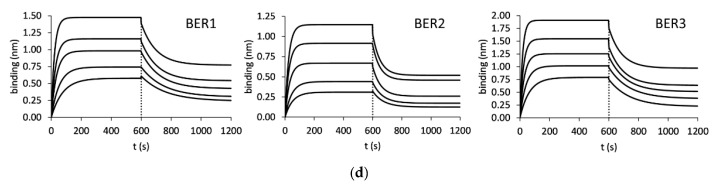
Anti-CD59 antigen-binding Fc fragments (Fcabs) from initial selections of Fcab libraries: (**a**) HPLC-SEC chromatography of derived mAb^2^ molecules in native conditions (MWS: molecular weight standard); (**b**) ELISA with coated CD59; (**c**) Competition of biotinylated HuMax-20 (HX)-BER3 mAb^2^ with HX-BER1, HX-BER2 and HX-BER3; (**d**) Biolayer interferometry measurement of response to immobilized CD59: HX-BER1 to 3 were applied in 2-fold dilution steps starting from 1 µM. RX: rituximab, TRA: trastuzumab.

The antigen for selections was refolded from *E. coli* inclusion bodies at a final yield of 1.3 mg/L bacterial culture and was shown to react with a CD59-reactive antibody MEM-43 in enzyme-linked immunosorbent assay (ELISA) (Supplementary Figure S2). After biotinylation, it was applied for sorting of the Fcab library, which proceeded with an initial magnetic-activated cell sorting (MACS)-based selection and several sequential fluorescence-activated cell sorting (FACS)-based selections until the visible enrichment of antigen-positive clones. A fluorescently labelled structure-dependently binding anti-CH2 domain antibody was employed as a reporter of correct folding and normalization of the copy numbers of displayed Fcabs. No enrichment of antigen-binding clones could be obtained by sorting the library AB01EF01, even after 7 consecutive sorting rounds. In contrast, for AB03EF01, the enrichment of antigen-positive clones was apparent after 5 sorting rounds and for AB03EF03 after 4 sorting rounds (selection and affinity maturation strategy in Figure 1b). Sequencing of around 100 single colonies revealed 2 different Fcab sequences from each library and the clones were named BER1-4 (all antibody sequences in Supplementary Table S1). For further testing, they were expressed in immunoglobulin G1 (IgG1) mAb^2^ format with rituximab (RX) variable regions, HuMax-20 (ofatumumab) (HX) variable regions and trastuzumab (TRA) variable regions. For all antibodies a level of expression, similar as the parental antibody, was found. While all BER4-derived mAb^2^ molecules were delayed in HPLC, indicating a degree of interaction with the column matrix, other clones exhibited a profile similar as unmodified antibodies (Figure 2a). Their EC_50_ for the reactivity with passively coated CD59 in ELISA was determined to be around 100 nM (0.94 nM for MEM-43 antibody, used as a positive control) (Figure 2b). None of the clones has shown cross-reactivity with control antigens mouse serum albumin (MSA), ribonuclease A (RNase) and lysozyme, except for BER4 with lysozyme at the highest applied 1 µM-concentration, and this clone was therefore eventually excluded from further testing. At this point, a competition experiment in ELISA format was performed: while there was no competition of BER2 with BER3, BER1 and BER3 appeared to recognize an overlapping epitope on CD59 (Figure 2c). Further, the affinity of the clones was evaluated with biolayer interferometry (BLI). With immobilized antigen and the antibody in solution, the K_D_ for clones BER1 and BER3 was approximately 80 nM and for BER2 285 nM, and for all three clones the binding kinetics was of fast-on and fast-off rate (Figure 2d and Supplementary Table S2). 

In the next step, the ability of the clones to enhance complement-dependent cytotoxicity (CDC) was evaluated. We have chosen three cell lines, expressing different surface levels of CD20 and CD59, and one CD20-negative/CD59-positive cell line, and determined the numbers of the expressed molecules per cell using QIFIKIT^®^ (Table 1). CDC effect of RX, HX and the control anti-fluorescein antibody 4420 was also measured (Supplementary Figure S3).

**Figure 3:**
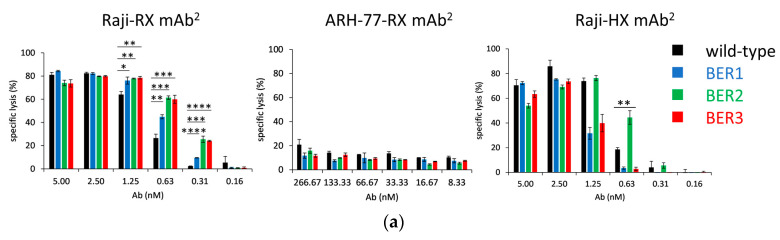

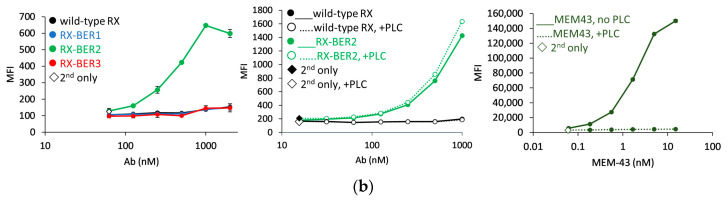
Cell assays with anti-CD20/anti-CD59 bispecific antibodies: (**a**) Complement-dependent cytotoxicity assay in rituximab (RX)-(left) and HuMax-20 (HX)-(right) format on Raji cells and RX-based mAb^2^ on ARH-77 cells (center). Sample size was *n* = 3 for Raji and *n* = 2 for ARH-77 cells. One-way ANOVA was used to determine the significance (* 0.01 ≤ *p* < 0.05, ** 0.001 ≤ *p* < 0.01, *** 0.0001 ≤ *p* < 0.001, **** *p* ≤ 0.0001); (**b**) SK-BR-3 cell staining of RX-BER mAb^2^ molecules (left panel), the interaction of RX-BER2 and RX with SK-BR-3 cells with and without phospholipase C (PLC)-treatment (central panel), MEM-43-staining of SK-BR-3 cells with and without PLC treatment (right panel). 2nd only: cells stained with fluorescent conjugate only, without primary antibody. Presented are mean and S.D.

All RX-based bispecific antibodies could elicit a stronger CDC effect on Raji cells comparing with the parental antibody at the concentrations lower than 1.25 nM, and BER3 was the most potent one, with an EC_50_ of 0.29 nM about-2-fold stronger than the parental antibody (0.63 nM) and the treatment caused up to twice the number of lysed cells (Figure 3a, left panel). There was no enhancement of the CDC on ARH77 cell line (Figure 3a, central panel). All tested bispecific antibodies were also CDC-competent when combined with HX-variable domains, but the effect of the parental antibody was not potentiated, except for BER2 at 0.63 nM (Figure 3a, right panel).

We were then interested to visualize cell surface binding of the anti-CD59 clones. Of RX-based bispecific antibodies, only BER2-mAb^2^ was able to bind to the surface of SK-BR-3 cells (Figure 3b). However, this interaction persisted after phospholipase C (PLC)-cleavage of the GPI-linked CD59, which prevented the binding of the control antibody MEM-43 (Figure 3b), so we assumed that the observed interaction signal was not specific.

**Figure 4:**
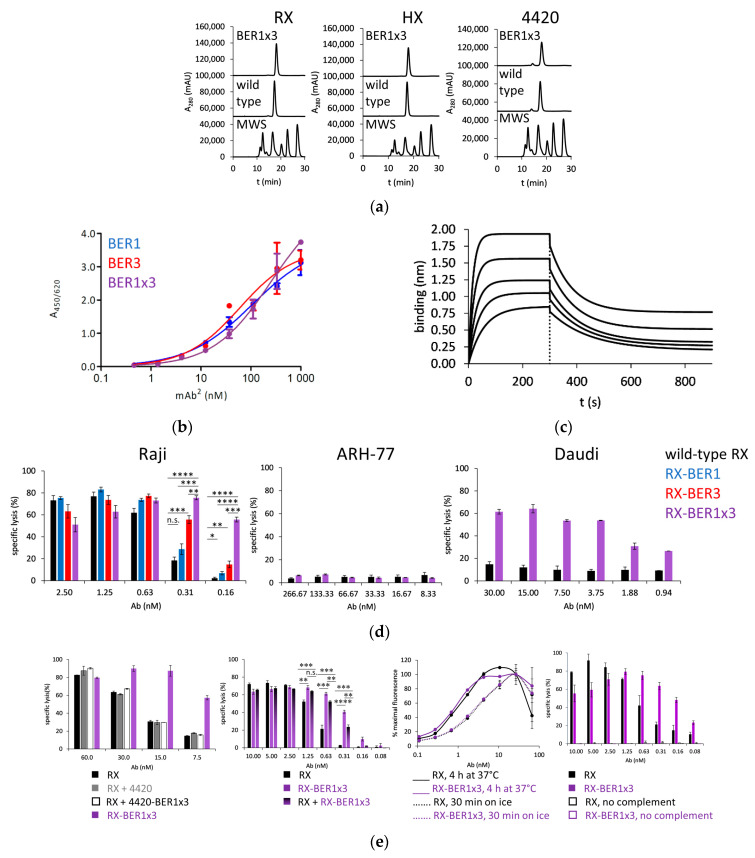
Characterization of shuffled BER1x3-clone based mAb^2^ molecules: (**a**) SEC-HPLC profiles in native conditions of rituximab (RX)-, HuMax-20 (HX)- and 4420-based mAb^2^ molecules (MWS: molecular weight standard); (**b**) ELISA response of RX-based bispecific antibodies with parental Fcabs and the shuffled clone to immobilized CD59; (**c**) Biolayer interferometry-determined binding of RX-BER1x3 to immobilized CD59, antibody in 2-fold dilutions starting with 300 nM; (**d**) Complement-dependent cytotoxicity (CDC) effect of RX-BER1, -3 and -1x3 for Raji and RX-BER1x3 for ARH-77 and Daudi cells. Sample size was *n* = 3 for Raji and *n* = 2 for ARH-77 and Daudi cells. One-way ANOVA was used to determine the significance (n.s. (not significant) 0.05 ≤ *p*, * 0.01 ≤ *p* < 0.05, ** 0.001 ≤ *p* < 0.01, *** 0.0001 ≤ *p* < 0.001, **** *p* ≤ 0.0001), mean and S.D. are presented; (**e**) 1st panel: CDC effect on Raji cells of RX, mix of RX and 4420 antibody, mix of RX and 4420-BER1x3 mAb^2^ and RX-BER1x3 mAb^2^ (*n* = 2), 2nd panel: CDC effect on Raji cells of RX, RX-BER1x3, and mix of RX and RX-BER1x3 (*n* = 3). One-way ANOVA was used to determine the significance (n.s. (not significant) 0.05 ≤ *p*, ** 0.001 ≤ *p* < 0.01, *** 0.0001 ≤ *p* < 0.001, **** *p* ≤ 0.0001), 3rd panel: Raji cell staining with RX and RX-BER1x3 at 4 °C and at 37 °C (*n* = 2), 4th panel: the effect of RX and RX-BER1x3 on Raji cells with and without complement (*n* = 2). Mean and S.D. are presented.

Seeing the CDC-potentiating activity of BER1 and BER3 clones, we decided to examine if shuffling of their AB- and EF-loops could enhance this effect. While the construct with BER3 AB-loop and BER1 EF-loop did not express well, BER1x3 Fcab gave rise to well-expressed bispecific antibodies with monomeric SEC-profile similar as the parental antibodies RX, HX or the control “silent” anti-fluorescein antibody 4420 (Figure 4a). In ELISA the shuffled clone gave a similar response as the parental clones (Figure 4b) and in BLI its affinity towards the CD59 was 71.9 nM, similar as measured for both parental clones (Figure 4c and Supplementary Table S2), but in the CDC assay with Raji cells in the RX-based mAb^2^ format it incited a stronger increase in specific cell lysis comparing with the parental clones (Figure 4d, left panel).

To inspect the dependency of RX-BER1x3 activity on the presence of both antigens on target cells, its effect on low-CD20 ARH-77 cells was tested and no significant difference to unmodified RX could be measured (Figure 4d, central panel). On the other hand, for Daudi, another CD20-positive cell line, up to 4-fold percentage of lysed cells was found comparing with the wild-type RX (right panel). A further experiment was done where the 4420-BER1x3 mAb^2^ was used in the CDC assay with Raji cells as a mixture with parental RX antibody (Figure 4e, 1st panel). Again, no difference to RX alone could be established. To examine if the potentiated CDC activity is really dependent on binding of CD20, we performed the CDC assay with the wild type RX, RX-BER1x3 and their equimolar mixture. The resulting EC_50_ values were at 0.88, 0.27 and 0.39 nM, respectively, and also the level of specific cytotoxicity caused by the mixture was between the wild-type antibody and the mAb^2^ (Figure 4e, 2nd panel).

Further, we were interested if RX-BER1x3 can bind better than RX to the surface of the Raji cells, but their reactivity with cellular surface in FACS was very similar at 4 °C as well as after the incubation at 37 °C, which are the typical conditions applied in the CDC assay (Figure 4e, 3rd panel). Neither RX nor the RX-BER1x3 could induce any cytotoxicity without added complement (Figure 4e, 4th panel).

**Figure 5:**
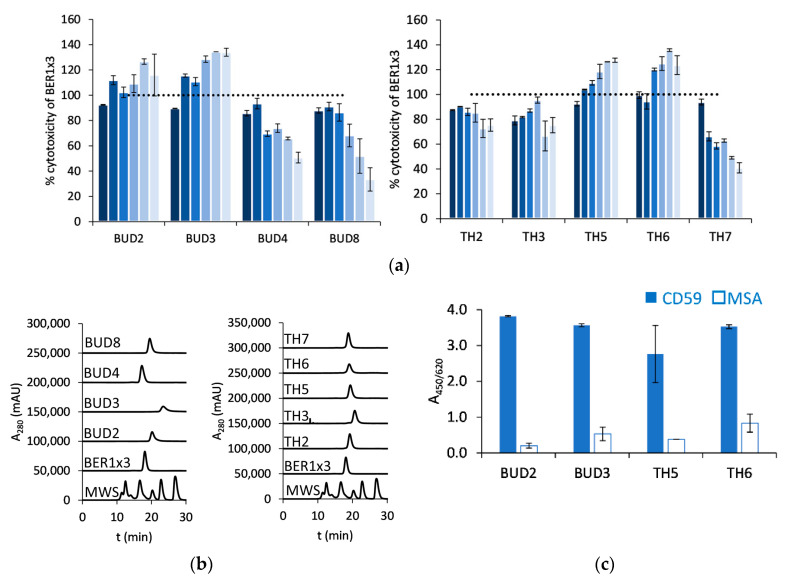
Affinity-matured BER1x3-derived clones from libraries BER1x3_4NNK (“BUD” clones) and BER1x3_5NNK (“TH” clones): (**a**) Complement-dependent cytotoxicity effect of rituximab (RX)-based mAb^2^ molecules on Raji cells, compared with parental RX-BER1x3, in 2-fold dilutions starting from 30 nM, decreasing concentrations are presented from dark to light blue (sample size *n* = 2, mean and S.D. are presented); (**b**) HPLC-SEC in native conditions of RX-based mAb^2^ molecules (MWS: molecular weight standard); (**c**) ELISA with CD59 and mouse serum albumin as a control antigen (sample size *n* = 2, mean and S.D. are shown).

We were wondering if by increasing antigen affinity the activity of the BER1x3—based mAb^2^ molecules can be further improved. As highly functional clones appeared to share the mutated residues in the EF loop, a further attempt of affinity maturation was taken to optimize the residues in the AB loop. We constructed and selected two pool expansion libraries with 4 (as in BER 1) or 5 (as in BER 3) randomized amino acid residues preceding the two tyrosines in AB loop, common to both clones. Resulting selected Fcab clones were tested for their activity in the CDC assay in RX-mAb^2^ format with Raji cells (Figure 5a) and then examined for their high-performance liquid chromatography—size exclusion chromatography (HPLC-SEC) profile (Figure 5b). BUD2, BUD3, TH5 and TH6 appeared to kill 20–30% more target cells than the parental clone, but all eluted with a delay in HPLC-SEC and showed undesired reactivity with MSA as a control antigen (Figure 5c).

We further aimed to possibly improve the activity of anti-CD59 clones by enhancing their recognition of mammalian cells-expressed antigen. For this purpose, we have expressed CD59 in HEK293-6E cells and purified it via his-tag and SEC (Supplementary Figure S2A). High amounts of dimeric species after His-based purification allowed us to determine the reactivity of commercially acquired CD59-specific antibodies also to this SEC-isolated oligomer (Supplementary Figure S2B,C). There was no reactivity of the monoclonal MEM-43 antibody with the dimeric version of mammalian cells-expressed CD59, which was only recognized by the polyclonal reagent. This indicates its substantial misfolding during the expression process. On the other hand, MEM-43 as well as the polyclonal antibody recognized both refolded monomer from bacteria and HEK293-6E-produced monomeric CD59. 

**Figure 6:**
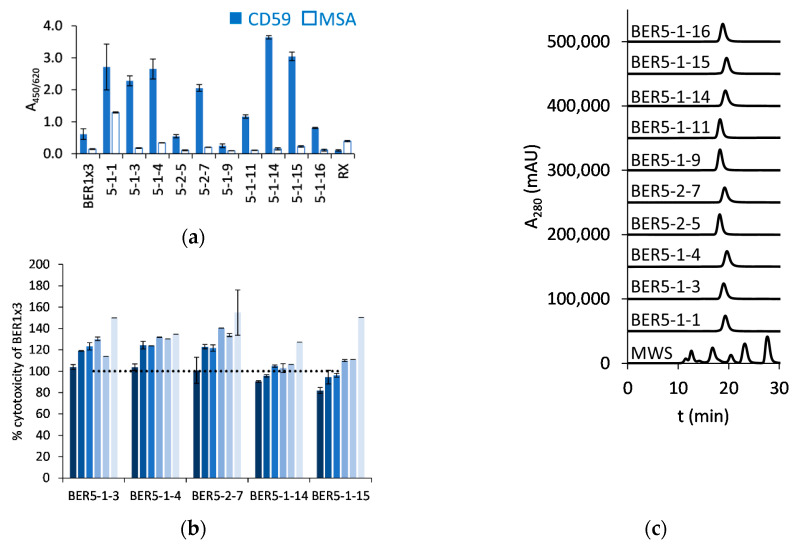

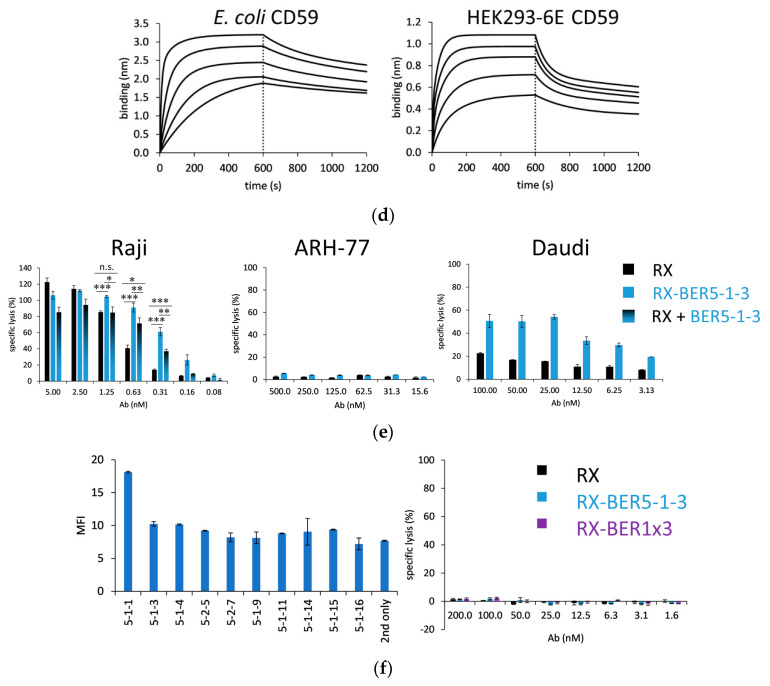
BER1x3-derived clones from libraries BER1x3_4NNK and BER1x3_5NNK, selected with mammalian cells-derived CD59 antigen: (**a**) ELISA response of rituximab (RX)-based mAb^2^ molecules to HEK-expressed CD59 and mouse serum albumin as a control antigen; (**b**) Complement-dependent cytotoxicity (CDC) assay of RX-based bispecific antibodies on Raji cells, compared with parental clone RX-BER1x3, in 2-fold dilutions starting from 5 nM, decreasing concentrations are presented from dark to light blue (sample size *n* = 2, mean and S.D. are shown); (**c**) Profiles of RX-derivates in SEC-HPLC in native conditions (MWS: molecular weight standard); (**d**) Biolayer interferometry-measured response of RX-BER5-1-3 to bacterially expressed and refolded CD59 (left panel) and mammalian cells expressed CD59 (right panel), in 2-fold dilutions starting from 300 nM; (**e**) Comparison of the CDC effect of RX and RX-BER5-1-3 on Raji cells (left panel), ARH-77 cells (central panel) and Daudi cells (right panel) (sample size *n* = 3 for experiments done with Raji and *n* = 2 for ARH-77 and Daudi, one-way ANOVA was used to determine the significance (n.s. (not significant) 0.05 ≤ *p*, * 0.01 ≤ *p* < 0.05, ** 0.001 ≤ *p* < 0.01, *** 0.0001 ≤ *p* < 0.001), mean and S.D. are shown; (**f**) SK-BR-3 cell surface binding of RX-based mAb^2^ molecules at 2 µM concentration (left panel) and the CDC (in)activity of RX and the mAb^2^ derivates on this cell line, sample size *n* = 2 (right panel). Mean and S.D. are presented.

After the selection of the pool expansion-libraries based on BER1x3 with mammalian cells-expressed monomeric antigen, the variants with 4 randomized residues did not enrich, but from the other library clones were discovered that recognized this antigen variant also in soluble format (Figure 6a). Strongly binding candidates were tested in RX-framework in a CDC assay with Raji cells (BER5-1-1 was excluded due to a cross-reactivity with the control antigen MSA). Three of the five tested clones achieved 20–40% more specific lysis than the parental BER1x3 (Figure 6b), and we characterized BER5-1-3 further as the HPLC-SEC profiles of BER5-1-4 and BER5-2-7 showed delayed elution (Figure 6c). The affinity of BER5-1-3 for the mammalian cell-expressed antigen was 40 nM and interestingly, also the affinity to the bacterial refolded CD59 (2.8 nM) was improved in comparison with the parental clone (Figure 6d and Supplementary Table S2). In comparison with RX, the EC_50_ in the CDC with Raji cells improved from 0.88 nM to 0.28 nM, which was very similar to values obtained with the parental mAb^2^ RX-BER1x3. The increase of specific cytotoxicity was maximally 4-fold more than with wild-type RX (Figure 6e, left panel). There was not any measurable toxicity for the ARH-77 cells (Figure 6e, central panel), but up to 2.5-fold more Daudi cells were killed with the mAb^2^ than with RX (Figure 6e, right panel). We also here tested the dependency on the CD20 binding for the CDC activity by exposing the Raji cells to an equimolar mixture of RX and RX-BER5-1-3, and the EC_50_ was between those measured for each of the two antibodies when applied alone (0.36 nM), with the level of specific cytotoxicity intermediate between single agents (Figure 6e, left panel). The binding to the strongly CD59-positive SK-BR-3 cells of all tested clones was at the background level (except for the clone BER5-1-1 due to its sticky character) (Figure 6f, left panel) and neither RX nor its derivate mAb^2^ molecules BER1x3 and BER5-1-3 had any CDC effect on these cells (Figure 6f, right panel), showing that their activity is dependent on CD20 interaction.

## 3. Discussion

In this work, we aimed to construct bispecific antibodies which can bind to an antigen, typically overexpressed on lymphoma cells, such as CD20, and simultaneously interact with CD59 to enhance the CDC for target-overexpressing cells. We have used a symmetric bispecific format mAb^2^ and isolated CD59-specific Fcab clones, which were then affinity matured to single-digit nanomolar affinity towards the novel cognate antigen. The two different antigen forms used for the selections were either expressed and refolded from bacterial inclusion bodies or harvested from the supernatant of HEK293 and they differed substantially in the degree of glycosylation. Using mutagenesis studies, it has been previously shown that N-glycosylation is not required for the maintenance of native-like fold and complement-inhibitory activity of CD59, when expressed on the cell surface [32,33]. Nevertheless, the N-linked glycan is believed to keep the extracellular domain of cell-bound CD59 suitably positioned for a strong interaction with MAC, and indeed the non-glycosylated protein loses most of its activity on the erythrocyte membrane [34]. However, novel investigations into N-glycan-deficient CD59 have revealed that enhanced cellular stress results in abundant inositol-acylated GPI-anchored misfolded protein variants at the cell surface [35], and it was also possible to achieve an enhanced CDC resistance by recombinant surface expression of non-glycosylated CD59 [36].

The anti-CD59 clones identified in this study were not able to block the effect of CD59 as stand-alone antibodies with “silent” Fab arms when they were applied as a mixture with rituximab. The activity of the bispecific CD20/CD59-reactive antibodies was strictly dependent on the presence of CD20 on the cell surface and they had no effect on the CD20 low-expressing or non-expressing cells. Even with the high antigen affinity variants, binding to the surface of the CD59-overexpressing cells was not detected. There was also no difference in cell surface reactivity between wild-type rituximab and rituximab-anti-CD59 antibody. It was also interesting to see that after selecting anti-CD59 variants that could react with HEK-expressed antigen, despite the concurrent increase in affinity towards the bacterially expressed CD59, the improvement of the activity in CDC-mediated lysis amounted only to about 10% more lysed cells comparing with the parental antibody, and no change in EC_50_ was achieved. In the process of complement blockade, CD59 undergoes a substantial structural rearrangement, and it is possible that the antibodies react preferentially with such forms. Rituximab and ofatumumab induce the translocation of CD20 to lipid rafts [37], and antigen accumulation allows a critical concentration of immune complexes required for the binding of the C1q component [38]. The interaction of the bispecific antibody with another membrane antigen, such as CD59, could influence the mobility of the primary CD20 target molecule and enhance antibody-dependent complement activation.

It has been previously shown that CDC mediated by rituximab depends on the expression level of CD20 [39], but the lack of such effect in refractory cell lines, such as ARH-77, has been attributed not only to somewhat lower levels of CD20, but also to higher expression of mCRPs, such as CD59 and CD55 [40,41]. The expression and ratio of CD20 and CD59 molecules vary substantially in non-Hodgkin’s lymphoma and chronic lymphocytic leukemia patient-derived cell lines [42,43,44] and it will be extremely interesting to discover, to what extent the novel anti-CD20/CD59 construct can enhance CDC effect of RX for such diverse spectrum of cells. These results will be the starting point for design of an in vivo study, where the enhanced tumor killing potency of the bispecific construct could shorten the required time of therapy with RX and diminish the probability of downregulation of CD20, which has been observed in cell lines as well as identified as a common cause of RX resistance in patients [45,46]. 

The RX-anti-CD59 bispecific antibodies increased the number of complement-lysed cells, and a lower concentration was required for their half-maximal effect. This is valuable as the CDC effect could be at a saturation level at lower concentration of the antibodies, and a lower quantity of complement components would be consumed, which can be a limiting factor for excessively applied antitumor mAbs [47,48]. With the current development of trispecific antibodies, including such based on mAb^2^ format as used here [49], it will be very interesting to see if the simultaneous interaction with another CRP, such as CD55, can further potentiate the CDC activity of CD20-targeting antibodies.

## 4. Materials and Methods

### 4.1. Antigen Preparation: Expression, Refolding, Purification and Biotinylation

For bacterial expression, CD59-encoding sequence (synthetized by Geneart (Regensburg, Germany)) was amplified using primers CD59nde1 and CD59bam2 (all primer sequences in the Supplementary Table S3, primers were synthesized by Microsynth, Balgach, Switzerland) and cloned to pET28a vector (Merck Millipore, Burlington, MA, USA), linearized with *Nde*I and *Bam*HI (all restriction enzymes were from New England Biolabs, Ipswitch, MA, USA). After transformation to *E. coli* BL21(DE3) (Merck Millipore, Burlington, MA, USA), plasmid was sequenced for the presence of the correct expression cassette. *E. coli* transformants were cultured in 500 mL M9ZB medium with 2% glucose and 50 μg/mL kanamycin at 30 °C to an OD_600_ of 1 and induced in the same volume of M9ZB with 1% glycerine, 50 μg/mL kanamycin and 1 mM isopropyl-β-D-thiogalactopyranosid (IPTG) at 16 °C overnight (all media components were from Merck Millipore, Burlington, MA, USA). This culture with an OD_600_ value about 5.0 was distributed to 50-mL-tubes in 30-mL aliquots and centrifuged at 1 300× *g* for 15 min at 4 °C. The supernatant was discarded, and each pellet resuspended in 30 mL 50 mM Tris with 0.1 M NaCl and 1 mM ethylenediaminetetraacetic acid (EDTA), pH 8.0 (all chemicals were from Merck Millipore, Burlington, MA, USA, unless stated otherwise). Sonication was performed using Branson Sonifier 250 (Emerson Electrics, Brookfield, CT, USA)), with 12 sequential continuous pulses of 10 s at 20% duty cycle, each followed by a 30-s-incubation on ice. The aliquots were pooled to 2 × 240 mL and clarified with a centrifugation step at 7000× *g*, 20 min at 4 °C. Each of the two pellets was washed twice with 100 mL 10 mM Tris, 1% Triton-X, pH 8.0, at 7000× *g* for 20 min at 4 °C. Pellets were resuspended each in 50 mL 2 M urea, 25 mM Tris and 0.5 mM EDTA, pH 8.0, and 4 aliquots of 25 mL were transferred to 50-mL-tubes and sonicated with 3 continuous pulses of 10 s at 20% duty cycle. After centrifugation at 7000× *g* for 20 min at 4 °C, another wash step with 25 mL of the same buffer per aliquot was performed. Pellet was collected at 7000× *g* for 20 min at 4 °C, it was resuspended in 50 mL 8 M urea, 50 mM Tris, 1 mM EDTA, pH 8.0, with freshly added 5 mM dithiothreitol (DTT). Two 25-mL-aliquots were sonicated with three 5-s-continuous pulses at 20% duty cycle. Finally, the supernatant was harvested at 3000× *g* for 10 min at 4 °C, distributed to 10 mL aliquots and used immediately or stored at –80 °C. For refolding, a 10 mL aliquot was diluted into 500 mL of refolding buffer (50 mM Tris, 1 M NaCl, 1 mM reduced glutathione, 3 mM oxidized glutathione, 1 mM EDTA) at 4 °C and incubated overnight.

For purification, this sample was filtered through a 0.45-μm filter and loaded onto a phosphate-buffered saline (PBS)/0.5 M NaCl, pH 7.5-equilibrated 1-mL-HisTrap^™^ Excel column (Cytiva, Marlborough, MA, USA), at 1 mL/min. After washing with PBS/0.5 M NaCl, pH 7.5, and a PBS/0.5 M NaCl/20 mM imidazole pH 7.5—wash step to remove weakly bound material, the protein of interest was eluted with a 10-mL-gradient of 20 to 500 mM imidazole in PBS/0.5 M NaCl, pH 7.5, and 1-mL-fractions were collected. Fractions were analyzed using sodium dodecyl sulfate–polyacrylamide gel electrophoresis (SDS-PAGE) followed by Coomassie staining. Samples were mixed with Sample loading Dye (Thermo Fisher Scientific, Waltham, MA, USA) and loaded into the wells of Nu-PAGE 4–12% gels without further treatment. Gels were developed in 2-(N-morpholino) ethanesulfonic acid (MES) buffer (Thermo Fisher Scientific, Waltham, MA, USA) at 130 V for 35 min and proteins visualized using Novex Blue Colloidal Staining kit (Thermo Fisher Scientific, Waltham, MA, USA). Fractions containing the protein of interest were pooled and concentrated using Vivaspin^TM^ 20 centrifugation units (Merck Millipore, Burlington, MA, USA) with polyethersulfone membrane and molecular weight cut-off (MWCO) of 3000 Da, using centrifugation at 1000× *g* at 4 °C and PBS, pH 7.2 for buffer exchange. Protein concentration was determined using A_280_ determined with Nanodrop 2000c (Thermo Fisher Scientific, Waltham, MA, USA) and extinction coefficient of 1.074. The protein was stored at –80 °C until further use.

For the expression in mammalian cells, CD59-encoding sequence was amplified using primers CD59nhe1 and CD59bam2 and cloned to the vector pTT22SSP4 (Canadian National Research Council (CNRC), Ottawa, ON, Canada) using E. coli TOP10 cells (Thermo Fisher Scientific, Waltham, MA, USA). The desired plasmid sequence was verified by sequencing and used for transfection of HEK293-6E cells (CNRC, Ottawa, ON, Canada) following the protocol of the manufacturer. After 5 days of expression, the supernatant was clarified at 1000× *g* for 15 min at 4 °C and supplemented with PBS. Ni-nitrilotriacetic acid (NTA)-affinity chromatography was performed using Hi-Trap Excel column (Cytiva, Marlborough, MA, USA) equilibrated with PBS. After loading was completed with rinsing with PBS, weakly bound proteins were rinsed off the column with PBS with 20 mM imidazole and for elution, a gradient from 20 to 500 mM imidazole was applied in 5 column volumes. Eluted fractions were collected and analyzed using SDS-PAGE followed by Coomassie staining as described above. After pooling the relevant fractions, the buffer was exchanged for PBS, pH 7.2, as described above. The protein was quantified using A_280_ as determined by Nanodrop 2000c analysis (Thermo Fisher Scientific, Waltham, MA, USA) and the extinction coefficient of 1.194 and stored at –80 °C until use.

For both types of CD59 antigen, monomeric form was isolated by preparative size exclusion chromatography using a HiLoad 16/600 Superdex 75 pg column (Cytiva, Marlborough, MA, USA) and eluted with PBS at 1 mL/min. Typical final yield of monomeric final product was 1.2 mg/L culture for the *E. coli*-expressed CD59 and 1.0 mg/L HEK293-6E cells for the mammalian cell-derived material. 

Biotinylation of the antigen was performed using the EZ-link^TM^-NHS-LC-LC-biotinylation kit (Thermo Fisher Scientific, Waltham, MA, USA), exactly according to the manufacturer’s instructions, at a ratio of protein to biotin of 1:3. After 2-h-incubation with the biotinylation reagent at room temperature (RT), the reaction mix was dialysed against a 100-fold volume of PBS at 4 °C overnight, using 3 500 Da-MWCO dialysis tubing. The protein concentration was measured and the preparation was stored at –80 °C.

### 4.2. Yeast Libraries, Quality Control and Selections

Yeast Fcab libraries were constructed using gap repair-driven homologous recombination essentially as described before [50]. For the initial sorting, three large yeast libraries were constructed using primers listed in Supplementary Table S3 (synthesized by ELLA Biotech, Fürstenfeldbruck, Germany) to amplify the Fc-CH3 domain insert and transform it into *S. cerevisiae* EBY100 (Thermo Fisher Scientific, Waltham, MA, USA) together with *Bsm*BI-linearized pyd1-Fc dem vector [51]. Cultivation and induction were performed according to standard protocols [31]. The percentage of yeast cells binding to the anti-Xpress antibody (Thermo Fisher Scientific, Waltham, MA, USA), conformation-sensitive anti-human CH2 antibody (Bio-Rad, Hercules, CA, USA), protein A (Sigma-Aldrich, St. Louis, MO, USA) and CD64 (R&D Systems, Minneapolis, Minnesota, USA) was determined as described before [50]. The first antigen selection round proceeded with magnetic activated cell sorting (MACS) as described previously [52], using 1 μM biotinylated CD59, refolded from bacterial inclusion bodies, and further sorting rounds were performed with yeast cells stained with 1 μM biotinylated CD59 and a fluorescein isothiocyanate (FITC)-conjugated anti-human CH2 antibody (Bio-Rad, Hercules, CA, USA), diluted 1:200 in 10% bovine serum albumin (BSA)-PBS. Bound CD59 was detected using the conjugated streptavidin-Alexa Fluor^®^ 647 or NeutrAvidin^®^-phycoerythrin (PE) (both Thermo Fisher Scientific, Waltham, MA, USA), diluted 1:1000, interchangeably in the sorting rounds. At least the 20-fold output of the previous sorting round was processed using ARIA I instrument (Beckton Dickinson, Franklin Lakes, NJ, USA): top 0.1% of the double-positive yeast cells after excluding the doublets and autofluorescent cells were collected, cultivated, induced and further sorted until the visible enrichment of antigen-binding clones.

At this point, the output cells were plated out to solid medium to be able to screen single colonies. The sequences of plasmids of clones, positive for antigen binding, were determined. A Zymolyase (Zymo Research, Irvine, CA, USA) digest of a single colony in 30 μL Solution I (Zymoprep II Kit, Zymo Research, Irvine, CA, USA) was prepared according to manufacturer’s instructions and used as a template for PCR reaction with primers pydfwd and pydrev. Unique binders were cloned using CH3-domain exchange into pTT5 vector encoding the relevant antibody heavy chain sequence: RX, HX, TRA or 4420. Mutated CH3 domains were amplified from yeast lysate using Q5 Hi-Fi Polymerase (New England Biolabs, Ipswich, MA, USA) using primers ABnest and CH3sbam2 and cloned into the vectors with *Bsr*GI-*Bam*HI-excised wild-type CH3 sequence. After transformation to *E. coli* TOP10, plasmid sequences were controlled for correctness and the antibodies were expressed in HEK293-6E cells as described above.

Following the discovery of the first biologically active clones, shuffling of the AB- and EF-loop sequences was performed. For this purpose, the sequences of the loops were amplified using primers ABnest and 3CD for the AB- and 5EF and upnest for the EF-loop. Shuffled clones were constructed using EBY100 transformation with *Bsm*BI-linearized pyd1dem vector. Cloning and expression of the soluble bispecific antibodies proceeded as described above.

Pool expansion libraries were constructed using primers BER1x3_4NNK and BER1x3_5NNK and upnest primer, with the clone BER1x3 DNA as a template. PCR products were transformed together with the *Bsm*BI-linearized pyd1dx dem vector to EBY100. As these were relatively small libraries (theoretical size of 1.6 × 10^5^ and 3.2 × 10^6^), all selection rounds were performed with FACS. First the threshold antigen binding concentration was determined for the parental clone, and the library was stained with a 3-fold lower concentration. Anti-CH2-FITC antibody was used for normalization and the library was processed as described above, using 10-fold oversampling for the input of each sorting round.

### 4.3. Production and Biophysical Analysis of Bispecific Antibodies

The pTT5-based vectors (CNRC, Ottawa, ON, Canada) encoding the heavy and the light chain were mixed in the mass ratio of 1:1 and incubated with polyethyleneimine in the mass ratio of 1:2 in 1 mL F17 medium with 4 mM L-glutamine, 0.1% Pluronic F-68 and 25 μg/mL G-418 (all Thermo Fisher Scientific, Waltham, MA, USA) per μg DNA for 15 min at room temperature. The mix was added dropwise to 1 mL per µg DNA of HEK293-6E cells at a density of 1.5–2.0 x 10^6^ cells/mL. Cells were kept on an orbital shaker at 125 rpm, at 37° and 5% CO_2_ at 80% humidity. After 2 days, TN-1 trypton (Organotechnie, La Courneuve, France) was added to an end concentration of 0.5%, and the expression continued for following 2 days. Supernatant of the expressing cultures was harvested at 1000× *g*, 4 °C for 5 min and filtered through a 0.45 µm-filter before the affinity purification with a 1-mL-Protein A column (Cytiva, Marlborough, MA, USA) as described before [53]. Eluted and neutralized antibody containing fractions were dialysed overnight at 4 °C against a 100-fold volume of PBS in Snakeskin tubing with MWCO of 10 000 Da (Thermo Fisher Scientific, Waltham, MA, USA), the concentration was determined using Nanodrop 2000c instrument (Thermo Fisher Scientific, Waltham, MA, USA) and the preparation was stored at –80 °C.

Analytical SEC-HPLC was performed using Shimadzu LC-20 (Shimadzu, Kyoto, Japan) equipped with a Superdex 200 Increase 10/300 GL column (Cytiva, Marlborough, MA, USA), in PBS with 200 mM NaCl at a constant flow rate of 0.75 mL/min. For analysis, 20 µg protein at about 1 mg/mL were loaded. Molecular weight standards (Bio-Rad, Hercules, CA, USA) containing marker proteins of 670, 150, 44, 17 and 1.5 kDa in size, were used for column equilibration.

### 4.4. Antigen Affinity

#### 4.4.1. ELISA-Based Assays with Human- and Bispecific Antibodies

CD59 was coated to the wells of a 96-well Maxisorp plate (NUNC, Rochester, New York, USA) at 2 µg/mL in 100 µL PBS and incubated for 1 h at RT. After washing 3 times with 200 µL PBS and blocking with 5% BSA-PBS for 1 h at RT, 100 µL of the dilution series of antibody samples in 2.5% BSA-PBS was loaded and incubated for 1 h at RT. After washing 3 times with 200 µL PBS, bound antibody was detected with anti-human kappa-horseradish peroxidase (HRP) conjugate (Sigma-Aldrich, St. Louis, MO, USA), diluted 1:4000 in 2.5% BSA-PBS, for 45 min at RT. After washing 3 times with PBS, bound enzyme was detected by adding 100 µL 3,3′,5,5′-tetramethylbenzidine (TMB) (Sigma-Aldrich, St. Louis, MO, USA), the reaction was stopped by adding 100 µL 30% H_2_SO_4_ and the absorbance was read at 450/620 nm in Tecan Sunrise reader (Tecan, Männedorf, Switzerland). Duplicate measurements were taken. The results were evaluated with GraphPad Prism 5.0 (GraphPad Software, San Diego, CA, USA) to determine the EC_50_ of the compounds.

For the competition assay, the antibody HX-BER3 was biotinylated in the ratio protein to biotin of 1:3 as described above, and the competing antibodies were added at 2 µM and incubated at RT for 30 min before they were applied to the coated and blocked ELISA plate. For detection, streptavidin-HRP (Thermo Fisher Scientific, Waltham, MA, USA), diluted 1:2000 in 2% BSA-PBS, was used.

Control antigens for ELISA (lysozyme, MSA and RNase A (all Sigma-Aldrich, St. Louis, MO, USA)) were reconstituted in PBS and used for coating at 20 µg/mL.

#### 4.4.2. ELISA Assays with Control Antibodies

Monoclonal anti-CD59 antibody MEM43 antibody (ab9182, Abcam, Cambridge, UK) was detected with anti-mouse-HRP (Sigma-Aldrich, St. Louis, MO, USA), at a 1:2 500 dilution. Polyclonal anti-CD59 RP-02 antibody (12474-RP02, SinoBiological, Beijing, China) was detected with anti-rabbit-HRP (Sigma-Aldrich, St. Louis, MO, USA) at a dilution of 1:2 500 in 2.5% BSA-PBS.

#### 4.4.3. Biolayer Interferometry (BLI)

BLI was performed using Octet RED96 (Sartorius, Göttingen, Germany) at a constant temperature of 25 °C. The assay buffer was PBS, pH 7.4, with 1× Kinetics Buffer (Sartorius, Göttingen, Germany). Biotinylated CD59 was used for coating of super streptavidin biosensors (Sartorius, Göttingen, Germany) at 10 µg/mL for 300 s, and bispecific antibodies in 2-fold-dilution steps were allowed to bind for 600 s and dissociate for 600 s. Buffer-only controls were subtracted from the response curve and the kinetic parameters were derived with Octet Evaluation software 11.1 (Sartorius, Göttingen, Germany) using bivalent binding model.

### 4.5. Cell-Based Assays

#### 4.5.1. Cell Culture

Raji (CCL-86^™^), ARH-77 (CRL-1621^™^), Daudi (CCL-213^™^) and SK-BR-3 (HTB-30^™^) cells were purchased from ATCC (Manassas, VA, USA). Raji, ARH-77 and Daudi were propagated in RPMI with 10% fetal bovine serum (FCS) (both Sigma-Aldrich, St. Louis, MO, USA) with 4 mM glutamine, sodium pyruvate and penicillin/streptomycin (Thermo Fisher Scientific, Waltham, MA, USA) at 37 °C in humidified atmosphere with 5% CO_2_ and diluted three times a week to be kept below 1 × 10^6^ cells/mL. ARH-77 cells were detached with a cell-scraper (TPP, Trasadingen, Switzerland). SK-BR-3 cells were kept in DMEM (Thermo Fisher Scientific, Waltham, MA, USA) with 10% FBS with 4 mM glutamine and penicillin/streptomycin and passaged twice a week by trypsinization: the cells in a T75 flask were rinsed twice with 12 mL pre-warmed PBS, detached with 3 mL 0.1% Trypsin/0.05 M EDTA (Sigma-Aldrich, St. Louis, MO, USA) for 5 min at 37 °C, at 5% CO_2_ in humidified atmosphere, and collected in 9 mL DMEM with all additives with a centrifugation at 300× *g*, 5 min at RT before resuspension in fresh complete medium.

#### 4.5.2. Complement-Dependent Cytotoxicity Assay

Cells were counted and only used if their viability determined using Trypan Blue Exclusion Assay was over 95%. 30,000 Raji cells, 40,000 ARH-77 cells, or 30,000 Daudi cells per well in 25 µL were incubated with 50 µL of antibody dilutions in DMEM-high glucose without phenol red (Sigma-Aldrich, St. Louis, MO, USA) with 2% low IgG serum (Promega, Madison, WI, USA), 4 mM glutamine, sodium pyruvate and penicillin/streptomycin, and 25 µL of guinea-pig complement (Sigma-Aldrich, St. Louis, MO, USA), diluted 1:2.5 in the same medium, in 96-U-shaped well culture plates for 4 h at 37 °C in humidified atmosphere with 5% CO_2_. For the last 45 min, 10 µL of lysis reagent (a component of CytoTox 96^®^ Non-Radioactive Cytotoxicity Assay, Promega) were added to the control wells and the readings of these wells corresponded to total lysis. After centrifugation at 300× *g* for 5 min at RT, 50 µL of the supernatant were transferred to a fresh 96-F-well plate, 50 µL of lactose dehydrogenase substrate (Promega, Madison, WI, USA) were added and incubated for 30 min at RT. The reaction was quenched after 30 min by adding 50 µL stopping solution (Promega, Madison, WI, USA), 100 µL of the reaction mix were transferred to a fresh 96-F-well plate and the absorbance at 492/620 nm was read in a Tecan Sunrise spectrophotometer. Wells without cells were considered background, and wells with cells but without any antibody were used for to estimate spontaneous lysis. The values of background were subtracted before calculating the percent of specific cytotoxicity in experimental wells according to the equation: 100 x (A_492/620_ in experimental wells-A_492/620_ spontaneous lysis)/A_492/620_ in total lysis wells. Triplicate or duplicate measurements were recorded. The results were evaluated with GraphPad Prism 5.0 (GraphPad Software, San Diego, CA, USA) to determine the EC_50_ of the compounds.

For some assays (those presented in Figure 4e (left panel), Figure 5a and Supplementary Figure S3 (upper row, left panel)), a new batch of guinea pig complement was used, which influenced the EC_50_ of the measured antibodies, but all compared compounds were measured side-to-side in the same assay.

#### 4.5.3. FACS Assays of Cell Surface Binding

Cells were harvested with centrifuging at 300× *g* for 5 min at 4 °C or with trypsinization, diluted to a concentration of 100,000 cells/mL in ice-cold 2% BSA-PBS and incubated for 15 min on ice. One-hundred-µL aliquots were distributed to the wells of a 96-U-shaped-well plate and pelleted at 300× *g*, 5 min at 4 °C. Dilution series of antibodies in 2% BSA-PBS was used for staining for 30 min on ice and their binding was detected with 100 µL per well of anti-human kappa-FITC (Sigma-Aldrich, St. Louis, MO, USA) reagent, diluted 1:200 in ice-cold 2% BSA-PBS, for another 30 min on ice. Finally, the cells were pelleted at 300× *g* for 5 min, resuspended in 200 µL ice-cold PBS and kept on ice until analysis with Guava^®^EasyCyte^TM^ flow cytometer (Luminex, Austin, TX, USA). Duplicate samples were measured.

For the test of potential internalization, RX and RX-BER1x3 bispecific antibody were incubated with the target cells at 37 °C for 4 h in humidified atmosphere with 5% CO_2_ with no other assay modifications.

For monitoring of CD59 levels, control antibody MEM-43 was used. The binding of anti-CD59 monoclonal antibody MEM-43 was detected with anti-mouse-F(ab)_2_-FITC conjugate (Sigma-Aldrich, St. Louis, MO, USA), diluted 1:200 in 2% BSA-PBS. 

In certain assays, phospholipase C (PLC) treatment was used for enzymatic removal of GPI-linked CD59 from the cell surface: for this purpose, the cells were harvested by trypsinization or centrifugation, rinsed twice with 1 mL ice-cold PBS per 10^6^ cells at 300× *g*, 5 min at 4 °C), and incubated in 0.5 mL of PBS with 0.5 U phosphatidyl-inositol specific-PLC from *Bacillus cereus* (Thermo Fisher Scientific, Waltham, MA, USA) for 1 h at 37 °C. Cells were then rinsed twice with PBS, blocked with 2% BSA-PBS at 10^6^ cells/mL and the staining procedure proceeded as described above.

#### 4.5.4. Number of Cell Surface Bound Molecules

QIFIKIT^®^ (Agilent, Santa Clara, CA, USA) was used to determine the number of CD20 and CD59 molecules on the cell surface. After harvesting, 100,000 cells per sample were blocked in 5% BSA-PBS, for 30 min on ice, and then incubated with rituximab or anti-CD59 MEM-43 antibody at a saturation concentration in PBS for 1 h on ice. After centrifugation at 300× *g*, 5 min at 4 °C and 2 subsequent washes with 1 mL 0.1% BSA-PBS, samples were incubated with a goat anti-mouse IgG (H + L) Alexa Fluor Plus 488 highly cross absorbed antibody at 0.1 mg/mL (A327223, Thermo Fisher Scientific, Waltham, MA, USA) for 45 min. Calibration beads provided were blocked and stained with the conjugate exactly according to manufacturer’s instructions. The number of surface-bound molecules was calculated after the extrapolation of the calibration curve derived from the 5 populations of control beads with different density of receptors for the secondary antibody.

## Figures and Tables

**Table 1:** Number of CD20 and CD59 molecules per cell of Raji, ARH-77, Daudi, and SK-BR-3 cell lines determined with QIFIKIT^®^.

Cell Line	No. of CD20 Molecules	No. of CD59 Molecules
Raji	13,298	140,350
ARH-77	3413	118,065
Daudi	31,409	1407
SK-BR-3	-	785,596

## Data Availability

The data presented in this study are available in this article and the associated Supplementary Materials.

## Supplementary Materials

The following supporting information can be downloaded at: https://www.mdpi.com/article/10.3390/ijms23095208/s1.

## References

[1] Ricklin D., Lambris J.D. (2016). Complement therapeutics. Semin. Immunol..

[2] Merle N.S., Church S.E., Fremeaux-Bacchi V., Roumenina L.T. (2015). Complement system part I - molecular mechanisms of activation and regulation. Front. Immunol..

[3] Ricklin D., Hajishengallis G., Yang K., Lambris J.D. (2010). Complement: A key system for immune surveillance and homeostasis. Nat. Immunol..

[4] Preissner K.T., Podack E.R., Müller-Eberhard H.J. (1985). The membrane attack complex of complement: Relation of C7 to the metastable membrane binding site of the intermediate complex C5b-7. J. Immunol..

[5] Serna M., Giles J.L., Morgan B.P., Bubeck D. (2016). Structural basis of complement membrane attack complex formation. Nat. Commun..

[6] Bloch E.F., Knight E.M., Carmon T., McDonald-Pinkett S., Carter J., Boomer A., Ogunfusika M., Petersen M., Famakin B., Aniagolu J. (1997). C5b-7 and C5b-8 precursors of the membrane attack complex (C5b-9) are effective killers of E. coli J5 during serum incubation. Immunol. Invest..

[7] Tomlinson S., Taylor P.W., Morgan B.P., Luzio J.P. (1989). Killing of gram-negative bacteria by complement. Fractionation of cell membranes after complement C5b-9 deposition on to the surface of Salmonella minnesota Re595. Biochem. J.

[8] Hoover D.L., Berger M., Oppenheim M.H., Hockmeyer W.T., Meltzer M.S. (1985). Cytotoxicity of human serum for Leishmania donovani amastigotes: Antibody facilitation of alternate complement pathway-mediated killing. Infect. Immun..

[9] Koski C.L., Ramm L.E., Hammer C.H., Mayer M.M., Shin M.L. (1983). Cytolysis of nucleated cells by complement: Cell death displays multi-hit characteristics. Proc. Natl. Acad. Sci. USA.

[10] Li C.K. (1975). Proof of the one-hit mechanism of complement-induced lysis. Immunochemistry.

[11] Huang Y., Qiao F., Abagyan R., Hazard S., Tomlinson S. (2006). Defining the CD59-C9 binding interaction. J. Biol. Chem..

[12] Lockert D.H., Kaufman K.M., Chang C.P., Husler T., Sodetz J.M., Sims P.J. (1995). Identity of the segment of human complement C8 recognized by complement regulatory protein CD59. J. Biol. Chem..

[13] Husler T., Lockert D.H., Kaufman K.M., Sodetz J.M., Sims P.J. (1995). Chimeras of human complement C9 reveal the site recognized by complement regulatory protein CD59. J. Biol. Chem..

[14] Yu J., Dong S., Rushmere N.K., Morgan B.P., Abagyan R., Tomlinson S. (1997). Mapping the regions of the complement inhibitor CD59 responsible for its species selective activity. Biochemistry.

[15] Zhao X.J., Zhao J., Zhou Q., Sims P.J. (1998). Identity of the residues responsible for the species-restricted complement inhibitory function of human CD59. J. Biol. Chem..

[16] Huang Y., Smith C.A., Song H., Morgan B.P., Abagyan R., Tomlinson S. (2005). Insights into the human CD59 complement binding interface toward engineering new therapeutics. J. Biol. Chem..

[17] Wickham S.E., Hotze E.M., Farrand A.J., Polekhina G., Nero T.L., Tomlinson S., Parker M.W., Tweten R.K. (2011). Mapping the intermedilysin-human CD59 receptor interface reveals a deep correspondence with the binding site on CD59 for complement binding proteins C8 alpha and C9. J. Biol. Chem..

[18] Takeda J., Miyata T., Kawagoe K., Iida Y., Endo Y., Fujita T., Takahashi M., Kitani T., Kinoshita T. (1993). Deficiency of the GPI anchor caused by a somatic mutation of the PIG-A gene in paroxysmal nocturnal hemoglobinuria. Cell.

[19] Fishelson Z., Donin N., Zell S., Schultz S., Kirschfink M. (2003). Obstacles to cancer immunotherapy: Expression of membrane complement regulatory proteins (mCRPs) in tumors. Mol. Immunol..

[20] Gelderman K.A., Tomlinson S., Ross G.D., Gorter A. (2004). Complement function in mAb-mediated cancer immunotherapy. Trends Immunol..

[21] Ziegler M., Wang X., Lim B., Leitner E., Klingberg F., Ching V., Yao Y., Huang D., Gao X.M., Kiriazis H. (2017). Platelet-targeted delivery of peripheral blood mononuclear cells to the ischemic heart restores cardiac function after ischemia-reperfusion injury. Theranostics.

[22] Zhao W.P., Zhu B., Duan Y.Z., Chen Z.T. (2009). Neutralization of complement regulatory proteins CD55 and CD59 augments therapeutic effect of herceptin against lung carcinoma cells. Oncol. Rep..

[23] Macor P., Secco E., Mezzaroba N., Zorzet S., Durigutto P., Gaiotto T., De Maso L., Biffi S., Garrovo C., Capolla S. (2015). Bispecific antibodies targeting tumor-associated antigens and neutralizing complement regulators increase the efficacy of antibody-based immunotherapy in mice. Leukemia.

[24] Brinkmann U., Kontermann R.E. (2017). The making of bispecific antibodies. mAbs.

[25] Leung K.M., Batey S., Rowlands R., Isaac S.J., Jones P., Drewett V., Carvalho J., Gaspar M., Weller S., Medcalf M. (2015). HER2-specific Modified Fc Fragment (Fcab) induces antitumor effects through degradation of HER2 and apoptosis. Mol. Ther..

[26] Gaspar M., Pravin J., Rodrigues L., Uhlenbroich S., Everett K.L., Wollerton F., Morrow M., Tuna M., Brewis N. (2020). CD137/OX40 Bispecific Antibody Induces Potent Antitumor Activity that Is Dependent on Target Coengagement. Cancer Immunol. Res..

[27] Kraman M., Faroudi M., Allen N.L., Kmiecik K., Gliddon D., Seal C., Koers A., Wydro M.M., Batey S., Winnewisser J. (2020). FS118, a Bispecific Antibody Targeting LAG-3 and PD-L1, Enhances T-Cell Activation Resulting in Potent Antitumor Activity. Clin. Cancer Res..

[28] Lakins M.A., Koers A., Giambalvo R., Munoz-Olaya J., Hughes R., Goodman E., Marshall S., Wollerton F., Batey S., Gliddon D. (2020). FS222, a CD137/PD-L1 Tetravalent Bispecific Antibody, Exhibits Low Toxicity and Antitumor Activity in Colorectal Cancer Models. Clin. Cancer Res..

[29] Wozniak-Knopp G., Bartl S., Bauer A., Mostageer M., Woisetschläger M., Antes B., Ettl K., Kainer M., Weberhofer G., Wiederkum S. (2010). Introducing antigen-binding sites in structural loops of immunoglobulin constant domains: Fc fragments with engineered HER2/neu-binding sites and antibody properties. Protein Eng. Des. Sel..

[30] Boder E.T., Wittrup K.D. (1997). Yeast surface display for screening combinatorial polypeptide libraries. Nat. Biotechnol..

[31] Chao G., Lau W.L., Hackel B.J., Sazinsky S.L., Lippow S.M., Wittrup K.D. (2006). Isolating and engineering human antibodies using yeast surface display. Nat. Protoc..

[32] Bodian D.L., Davis S.J., Morgan B.P., Rushmere N.K. (1997). Mutational analysis of the active site and antibody epitopes of the complement-inhibitory glycoprotein, CD59. J. Exp. Med..

[33] Rother R.P., Zhao J., Zhou Q., Sims P.J. (1996). Elimination of potential sites of glycosylation fails to abrogate complement regulatory function of cell surface CD59. J. Biol. Chem..

[34] Ninomiya H., Stewart B.H., Rollins S.A., Zhao J., Bothwell A.L.M., Sims P.J. (1992). Contribution of the N-linked carbohydrate of erythrocyte antigen CD59 to its complement-inhibitory activity. J. Biol. Chem..

[35] Liu Y.S., Guo X.Y., Hirata T., Rong Y., Motooka D., Kitajima T., Murakami Y., Gao X.D., Nakamura S., Kinoshita T. (2018). N-Glycan-dependent protein folding and endoplasmic reticulum retention regulate GPI-anchor processing. J. Cell Biol..

[36] Yu J., Abagyan R., Dong S., Gilbert A., Nussenzweig V., Tomlinson S. (1997). Mapping the active site of CD59. J. Exp. Med..

[37] Cragg M.S., Morgan S.M., Chan H.T.C., Morgan B.P., Filatov A.V., Johnson P.W.M., French R.R., Glennie M.J. (2003). Complement-mediated lysis by anti-CD20 mAb correlates with segregation into lipid rafts. Blood.

[38] Wright J.K., Tschopp J., Jaton J.C., Engel J. (1980). Dimeric, trimeric and tetrameric complexes of immunoglobulin G fix complement. Biochem. J..

[39] Van Meerten T., Van Rijn R.S., Hol S., Hagenbeek A., Ebeling S.B. (2006). Complement-Induced Cell Death by Rituximab Depends on CD20 Expression Level and Acts Complementary to Antibody-Dependent Cellular Cytotoxicity. Clin. Cancer Res..

[40] Taylor R.P., Lindorfer M.A. (2020). How Do mAbs Make Use of Complement to Kill Cancer Cells? The Role of Ca^2+^. Antibodies.

[41] Ge X., Wu L., Hu W., Fernandes S., Wang C., Li X., Brown J.R., Qin X. (2011). rILYd4, a human CD59 inhibitor, enhances complement-dependent cytotoxicity of ofatumumab against rituximab-resistant B-cell lymphoma cells and chronic lymphocyticleukemia. Clin. Cancer Res..

[42] Golay J., Zaffaroni L., Vaccari T., Lazzari M., Borleri G.-M., Bernasconi S., Tedesco F., Rambaldi A., Introna M. (2000). Biologic response of B lymphoma cells to anti-CD20 monoclonal antibody rituximab in vitro: CD55 and CD59 regulate complement-mediated cell lysis. Blood.

[43] Glennie M.J., French R.R., Cragg M.S., Taylor R.P. (2007). Mechanisms of killing by anti-CD20 monoclonal antibodies. Mol. Immunol..

[44] Pawluczkowycz A.W., Beurskens F.J., Beum P.V., Lindorfer M.A., Van de Winkel J.G.J., Parren P.W.H.I., Taylor R.P. (2009). Binding of Submaximal C1q Promotes Complement-Dependent Cytotoxicity (CDC) of B Cells Opsonized with Anti-CD20 mAbs Ofatumumab (OFA) or Rituximab (RTX): Considerably Higher Levels of CDC Are Induced by OFA than by RTX. J. Immunol..

[45] Czuczman M.S., Olejniczak S., Gowda A., Kotowski A., Binder A., Kaur H., Knight J., Starostik P., Deans J., Hernandez-Ilizaliturri F.J. (2008). Acquirement of Rituximab Resistance in Lymphoma Cell Lines Is Associated with Both Global CD20 Gene and Protein Down-Regulation Regulated at the Pretranscriptional and Posttranscriptional Levels. Clin. Cancer Res..

[46] Smith M.R. (2003). Rituximab (monoclonal anti-CD20 antibody): Mechanisms of action and resistance. Oncogene.

[47] Stasiłojć G., Österborg A., Blom A.M., Okrój M. (2016). New perspectives on complement mediated immunotherapy. Cancer Treat. Rev..

[48] Beurskens F.J., Lindorfer M.A., Farooqui M., Beum P.V., Engelberts P., Mackus W.J.M., Parren P.W.H.I., Wiestner A., Taylor R.P. (2012). Exhaustion of cytotoxic effector systems may limit monoclonal antibody-based immunotherapy in cancer patients. J. Immunol..

[49] Natale V., Stadlmayr G., Benedetti F., Stadlbauer K., Rüker F., Wozniak-Knopp G. (2022). Trispecific antibodies produced from mAb 2 pairs by controlled Fab-arm exchange. Biol. Chem..

[50] Sádio F., Stadlmayr G., Stadlbauer K., Rüker F., Wozniak-Knopp G. (2019). Yeast surface display and cell sorting of antigen-binding Fc fragments. Methods Mol. Biol..

[51] Wozniak-Knopp G., Stadlmayr G., Perthold J.W., Stadlbauer K., Woisetschläger M., Sun H., Rüker F. (2017). Designing Fcabs: Well-expressed and stable high affinity antigen-binding Fc fragments. Protein Eng. Des. Sel..

[52] Sádio F., Stadlmayr G., Eibensteiner K., Stadlbauer K., Rüker F., Wozniak-Knopp G. (2020). Methods for Construction of Yeast Display Libraries of Four-Domain T-Cell Receptors. Methods Mol. Biol..

[53] Benedetti F., Stracke F., Stadlmayr G., Stadlbauer K., Rüker F., Wozniak-Knopp G. (2021). Bispecific antibodies with Fab-arms featuring exchanged antigen-binding constant domains. Biochem. Biophys. Rep..

