# Supplementary material for: Bispecific mAb2 Antibodies Targeting CD59 Enhance the Complement-Dependent Cytotoxicity Mediated by Rituximab
Source: Int J Mol Sci. 2022 May 6;23(9):5208. doi: 10.3390/ijms23095208 (PMC9103234; doi:10.3390/ijms23095208)
Supplement: Supplementary file 1 [file ijms-23-05208-s001.zip › Figure_S1.pdf]

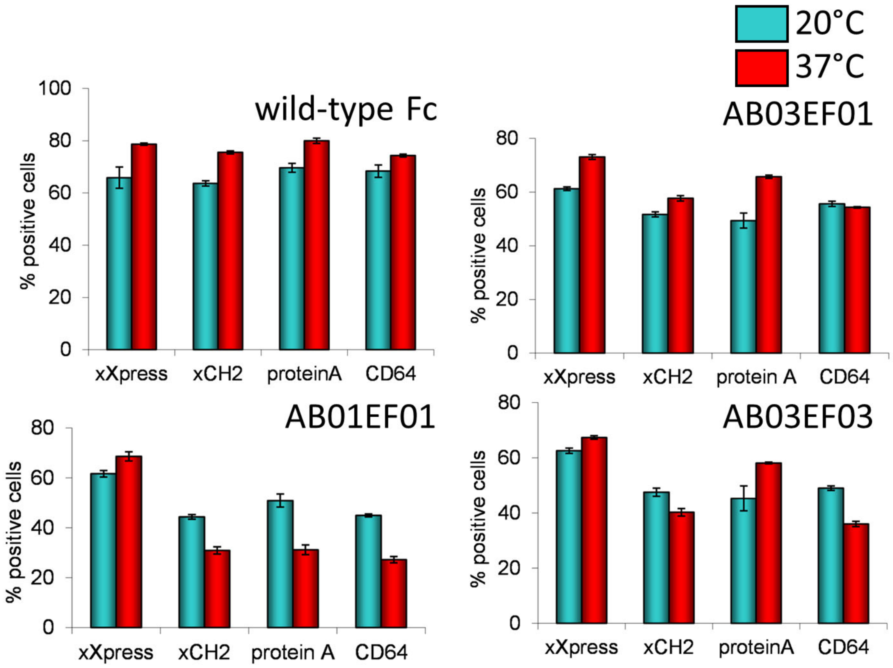

Supplementary Figure S1. Quality control of yeast display libraries: reactivity of yeast clones in display libraries, initially used for sorting, with anti-Xpress antibody, anti-CH2 antibody, protein A and CD64 was determined as percent of positive cells in the cultures induced at 20 and 37 °C. Triplicate measurements with mean and S.D. are presented.
