# Supplementary material for: Bispecific mAb2 Antibodies Targeting CD59 Enhance the Complement-Dependent Cytotoxicity Mediated by Rituximab
Source: Int J Mol Sci. 2022 May 6;23(9):5208. doi: 10.3390/ijms23095208 (PMC9103234; doi:10.3390/ijms23095208)
Supplement: Supplementary file 1 [file ijms-23-05208-s001.zip › Figure_S2.pdf]

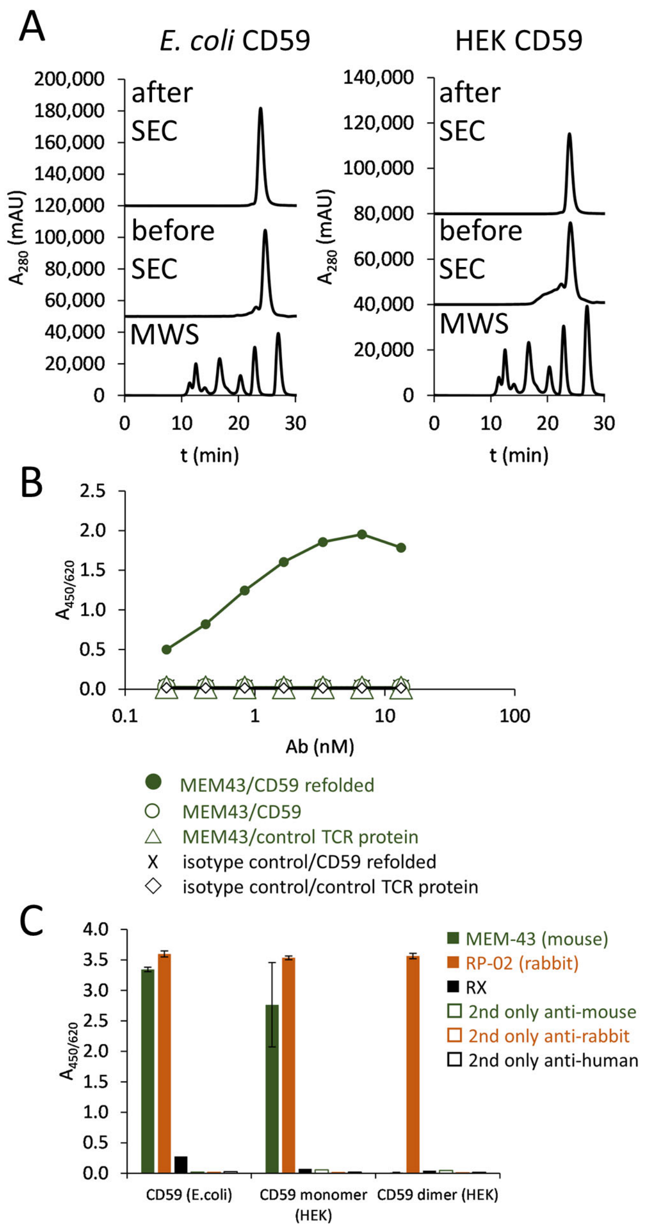

Supplementary Figure S2. CD59 antigen preparation: **(a)** Elution profile of bacterial and mammalian cells-expressed His-tag purified CD59 in HPLC-SEC in native conditions, before and after gel filtration (MWS: molecular weight standard); **(b)** binding of MEM-43 control monoclonal antibody to *E.coli*-derived antigen coated onto a Ni-NTA HisSorb plate (QIAgen, Hilden, Germany) at 2 μg/mL (TCR: soluble his-tagged T-cell receptor A6); **(c)** reactivity of monoclonal MEM-43, polyclonal RP-02 and RX to *E.coli* monomeric and HEK-cells expressed monomeric and dimeric antigen.
