# Supplementary material for: Bispecific mAb2 Antibodies Targeting CD59 Enhance the Complement-Dependent Cytotoxicity Mediated by Rituximab
Source: Int J Mol Sci. 2022 May 6;23(9):5208. doi: 10.3390/ijms23095208 (PMC9103234; doi:10.3390/ijms23095208)
Supplement: Supplementary file 1 [file ijms-23-05208-s001.zip › Figure_S3.pdf]

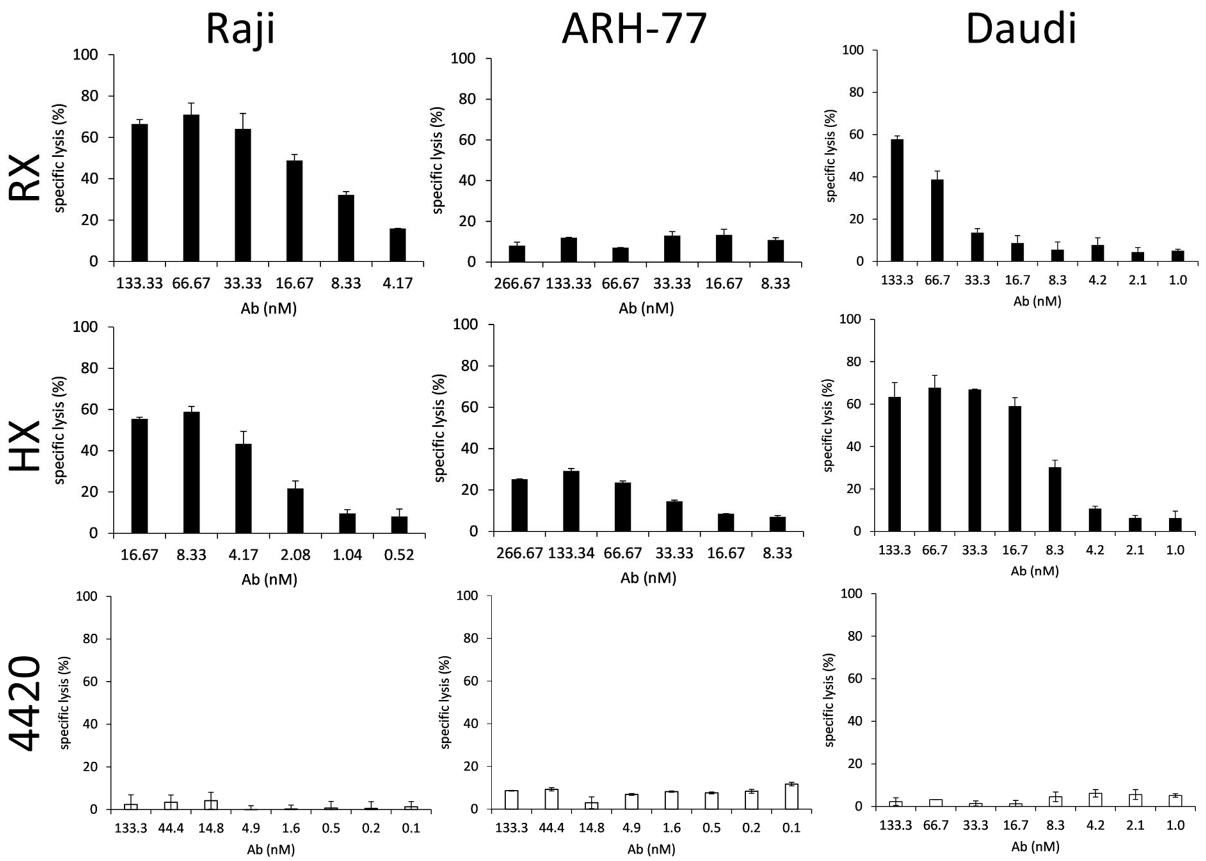

Supplementary Figure S3. The complement-dependent cytotoxicity effect of wild-type antibodies RX, HX and 4420 for Raji, ARH-77 and Daudi cells. The control antibody 4420 had no effect on any of the cell lines, while RX and HX incited potent lysis of Daudi cells with EC_{50} of 53.4 nM and 8.4 nM and Raji cells with EC_{50} of 9.4 and 2.6 nM. HX was also able to incite a low level of cytotoxicity for ARH-77 cells, with EC_{50} of 27.6 nM.
