# Supplementary material for: Bispecific mAb2 Antibodies Targeting CD59 Enhance the Complement-Dependent Cytotoxicity Mediated by Rituximab
Source: Int J Mol Sci. 2022 May 6;23(9):5208. doi: 10.3390/ijms23095208 (PMC9103234; doi:10.3390/ijms23095208)
Supplement: Supplementary file 1 [file ijms-23-05208-s001.zip › Table_S1.pdf]

Supplementary Table S1. Amino acid sequences of antibodies and antigens used in this study. The sequence of mutated residues in the AB loop of the CH3 domain of Fcab clones is in blue and EF in red.

Protein	Sequence
**Wild-type antibodies**	
**Rituximab (RX)**	
Heavy chain	QVQLQQPGAELVKPGASVKMSCKASGYTFTSYNMHWVKQTPGRGLEWIGAIYPGNGDTSYN<br>QKFKGKATLTADKSSSTAYMQLSSLTSEDSAVYYCARSTYYGGDWYFNVWGAGTTVTVSAA<br>STKGPSVFPLAPSSKSTSGGTAALGCLVKDYFPEPVTVSWNSGALTSGVHTFPAVLQSSGL<br>YSLSSVVTVPSSSLGTQTYICNVNHKPSNTKVDKKVEPKSCDKTHTCPPCPAPELLGGPSV<br>FLFPPKPKDTLMISRTPEVTCVVVDVSHEDPEVKFNWYVDGVEVHNAKTKPREEQYNSTYR<br>VVSVLTVLHQDWLNGKEYKCKVSNKALPAPIEKTISKAKGQPREPQVYTLPPSRDELTKNQ<br>VSLTCLVKGFYPSDIAVEWESNGQPENNYKTTPPVLDSDGSFFLYSKLTVDKSRWQQGNVF<br>SCSVMHEALHNHYTQKSLSLSPGK
Light chain	QIVLSQSPAILSASPGEKVTMTCRASSSVSYIHWFQQKPGSSPKPWIYATSNLASGVPVRF<br>SGSGSGTSYSLTISRVEAEDAATYYCQQWTSNPPTFGGGTKLEIKRTVAAPSVFIFPPSDE<br>QLKSGTASVVCLLNNFYPREAKVQWKVDNALQSGNSQESVTEQDSKDSTYSLSSTLTLSKA<br>DYEKHKVYACEVTHQGLSSPVTKSFNRGEC
**HuMax CD20 (HX)**	
Heavy chain	EVQLVESGGGLVQPGRSLRLSCAASGFTFNDYAMHWVRQAPGKGLEWVSTISWNSGSIGYA<br>DSVKGRFTISRDNAKKSLYLQMNSLRAEDTALYYCAKDIQYGNYYYGMDVWGQGTTVTVSS<br>ASTKGPSVFPLAPSSKSTSGGTAALGCLVKDYFPEPVTVSWNSGALTSGVHTFPAVLQSSG<br>LYSLSSVVTVPSSSLGTQTYICNVNHKPSNTKVDKKVEPKSCDKTHTCPPCPAPELLGGPS<br>VFLFPPKPKDTLMISRTPEVTCVVVDVSHEDPEVKFNWYVDGVEVHNAKTKPREEQYNSTY<br>RVVSVLTVLHQDWLNGKEYKCKVSNKALPAPIEKTISKAKGQPREPQVYTLPPSRDELTKN<br>QVSLTCLVKGFYPSDIAVEWESNGQPENNYKTTPPVLDSDGSFFLYSKLTVDKSRWQQGNV<br>FSCSVMHEALHNHYTQKSLSLSPGK
Light chain	EIVLTQSPATLSLSPGERATLSCRASQSVSSYLAWYQQKPGQAPRLLIYDASNRATGIPAR<br>FSGSGSGTDFTLTISSLEPEDFAVYYCQQRSNWPITFGQGTRLEIKRTVAAPSVFIFPPSD<br>EQLKSGTASVVCLLNNFYPREAKVQWKVDNALQSGNSQESVTEQDSKDSTYSLSSTLTLSK<br>ADYEKHKVYACEVTHQGLSSPVTKSFNRGEC
**Trastuzumab (TRA)**	
Heavy chain	EVQLVESGGGLVQPGGSLRLSCAASGFNIKDTYIHWVRQAPGKGLEWVARIYPTNGYTRYA<br>DSVKGRFTISADTSKNTAYLQMNSLRAEDTAVYYCSRWGGDGFYAMDYWGQGTLVTVSSAS<br>TKGPSVFPLAPSSKSTSGGTAALGCLVKDYFPEPVTVSWNSGALTSGVHTFPAVLQSSGLY<br>SLSSVVTVPSSSLGTQTYICNVNHKPSNTKVDKKVEPKSCDKTHTCPPCPAPELLGGPSVF<br>LFPPKPKDTLMISRTPEVTCVVVDVSHEDPEVKFNWYVDGVEVHNAKTKPREEQYNSTYRV<br>VSVLTVLHQDWLNGKEYKCKVSNKALPAPIEKTISKAKGQPREPQVYTLPPSRDELTKNQV<br>SLTCLVKGFYPSDIAVEWESNGQPENNYKTTPPVLDSDGSFFLYSKLTVDKSRWQQGNVFS<br>CSVMHEALHNHYTQKSLSLSPGK
Light chain	DIQMTQSPSSLSASVGDRVTITCRASQDVNTAVAWYQQKPGKAPKLLIYSASFLYSGVPSR<br>FSGSRSGTDFTLTISSLQPEDFATYYCQQHYTTPPTFGQGTKVEIKRTVAAPSVFIFPPSD<br>EQLKSGTASVVCLLNNFYPREAKVQWKVDNALQSGNSQESVTEQDSKDSTYSLSSTLTLSK<br>ADYEKHKVYACEVTHQGLSSPVTKSFNRGEC
**4420**	
Heavy chain	EVKLDETGGGLVQPGRPMKLSCVASGFTFSDYWMNWVRQSPEKGLEWVAQIRNKPYNYETY<br>YSDSVKGRFTISRDDSKSSVYLQMNNLRVEDMGIYYCTGSYYGMDYWGQGTSVTVSSASTK<br>GPSVFPLAPSSKSTSGGTAALGCLVKDYFPEPVTVSWNSGALTSGVHTFPAVLQSSGLYSL<br>SSVVTVPSSSLGTQTYICNVNHKPSNTKVDKKVEPKSCDKTHTCPPCPAPELLGGPSVFLF<br>PPKPKDTLMISRTPEVTCVVVDVSHEDPEVKFNWYVDGVEVHNAKTKPREEQYNSTYRVVS<br>VLTVLHQDWLNGKEYKCKVSNKALPAPIEKTISKAKGQPREPQVYTLPPSRDELTKNQVSL<br>TCLVKGFYPSDIAVEWESNGQPENNYKTTPPVLDSDGSFFLYSKLTVDKSRWQQGNVFSCS<br>VMHEALHNHYTQKSLSLSPGK

Light chain	DVVMTQTPLSLPVSLGDQASISCRSSQSLVHSNGNTYLRWYLQKPGQSPKVLIYKVSNRFS<br>GVPDRFSGSGSGTDFTLKISRVEAEDLGVYFCSQSTHVPWTFGGGTKLEIKRTVAAPSVFI<br>FPPSDEQLKSGTASVVCLLNNFYPREAKVQWKVDNALQSGNSQESVTEQDSKDSTYSLSST<br>LTLSKADYEKHKVYACEVTHQGLSSPVTKSFNRGEC
**Fcabs**<br>**Naïve selections**	
BER1	TCPPCPAPELLGGPSVFLFPPKPKDTLMISRTPEVTCVVVDVSHEDPEVKFNWYVDGVEVH<br>NAKTKPREEQYNSTYRVVSVLTVLHQDWLNGKEYKCKVSNKALPAPIEKTISKAKGQPREP<br>QVYTLPPSRDELLDGAYYNQVSLTCLVKGFYPSDIAVEWESNGQPENNYKTTPPVLDSDGS<br>FFLYSKLTVDQGRWMSGNVFSCSVMHEALHNHYTQKSLSLSPGK
BER2	TCPPCPAPELLGGPSVFLFPPKPKDTLMISRTPEVTCVVVDVSHEDPEVKFNWYVDGVEVH<br>NAKTKPREEQYNSTYRVVSVLTVLHQDWLNGKEYKCKVSNKALPAPIEKTISKAKGQPREP<br>QVYTLPPSRDELVGPQNYYNQVSLTCLVKGFYPSDIAVEWESNGQPENNYKTTPPVLDSDG<br>SFFLYSKLTVPYGRWYTGNVFSCSVMHEALHNHYTQKSLSLSPGK
BER3	TCPPCPAPELLGGPSVFLFPPKPKDTLMISRTPEVTCVVVDVSHEDPEVKFNWYVDGVEVH<br>NAKTKPREEQYNSTYRVVSVLTVLHQDWLNGKEYKCKVSNKALPAPIEKTISKAKGQPREP<br>QVYTLPPSRDELSQDMQYYNQVSLTCLVKGFYPSDIAVEWESNGQPENNYKTTPPVLDSDG<br>SFFLYSKLTVPQGRWYSGNVFSCSVMHEALHNHYTQKSLSLSPGK
BER4	TCPPCPAPELLGGPSVFLFPPKPKDTLMISRTPEVTCVVVDVSHEDPEVKFNWYVDGVEVH<br>NAKTKPREEQYNSTYRVVSVLTVLHQDWLNGKEYKCKVSNKALPAPIEKTISKAKGQPREP<br>QVYTLPPSRDELGPGMFWYNQVSLTCLVKGFYPSDIAVEWESNGQPENNYKTTPPVLDSDG<br>SFFLYSKLTVPQGRWYGGNVFSCSVMHEALHNHYTQKSLSLSPGK
**Loop shuffling**	
BER1x3	TCPPCPAPELLGGPSVFLFPPKPKDTLMISRTPEVTCVVVDVSHEDPEVKFNWYVDGVEVH<br>NAKTKPREEQYNSTYRVVSVLTVLHQDWLNGKEYKCKVSNKALPAPIEKTISKAKGQPREP<br>QVYTLPPSRDELLDGAYYNQVSLTCLVKGFYPSDIAVEWESNGQPENNYKTTPPVLDSDGS<br>FFLYSKLTVPQGRWYSGNVFSCSVMHEALHNHYTQKSLSLSPGK
BER3x1	TCPPCPAPELLGGPSVFLFPPKPKDTLMISRTPEVTCVVVDVSHEDPEVKFNWYVDGVEVH<br>NAKTKPREEQYNSTYRVVSVLTVLHQDWLNGKEYKCKVSNKALPAPIEKTISKAKGQPREP<br>QVYTLPPSRDELSQDMQYYNQVSLTCLVKGFYPSDIAVEWESNGQPENNYKTTPPVLDSDG<br>SFFLYSKLTVDQGRWMSGNVFSCSVMHEALHNHYTQKSLSLSPGK
**BER1x3_4NNK library selections – *E.coli* expressed CD59**	
BUD2	TCPPCPAPELLGGPSVFLFPPKPKDTLMISRTPEVTCVVVDVSHEDPEVKFNWYVDGVEVH<br>NAKTKPREEQYNSTYRVVSVLTVLHQDWLNGKEYKCKVSNKALPAPIEKTISKAKGQPREP<br>QVYTLPPSRDELRYRSYYNQVSLTCLVKGFYPSDIAVEWESNGQPENNYKTTPPVLDSDGS<br>FFLYSKLTVPQGRWYSGNVFSCSVMHEALHNHYTQKSLSLSPGK
BUD3	TCPPCPAPELLGGPSVFLFPPKPKDTLMISRTPEVTCVVVDVSHEDPEVKFNWYVDGVEVH<br>NAKTKPREEQYNSTYRVVSVLTVLHQDWLNGKEYKCKVSNKALPAPIEKTISKAKGQPREP<br>QVYTLPPSRDELRWRSYYNQVSLTCLVKGFYPSDIAVEWESNGQPENNYKTTPPVLDSDGS<br>FFLYSKLTVPQGRWYSGNVFSCSVMHEALHNHYTQKSLSLSPGK
BUD4	TCPPCPAPELLGGPSVFLFPPKPKDTLMISRTPEVTCVVVDVSHEDPEVKFNWYVDGVEVH<br>NAKTKPREEQYNSTYRVVSVLTVLHQDWLNGKEYKCKVSNKALPAPIEKTISKAKGQPREP<br>QVYTLPPSRDELRWRDYYNQVSLTCLVKGFYPSDIAVEWESNGQPENNYKTTPPVLDSDGS<br>FFLYSKLTVPQGRWYSGNVFSCSVMHEALHNHYTQKSLSLSPGK
BUD8	TCPPCPAPELLGGPSVFLFPPKPKDTLMISRTPEVTCVVVDVSHEDPEVKFNWYVDGVEVH<br>NAKTKPREEQYNSTYRVVSVLTVLHQDWLNGKEYKCKVSNKALPAPIEKTISKAKGQPREP<br>QVYTLPPSRDELQYRSYYNQVSLTCLVKGFYPSDIAVEWESNGQPENNYKTTPPVLDSDGS<br>FFLYSKLTVPQGRWYSGNVFSCSVMHEALHNHYTQKSLSLSPGK
**BER1x3_5NNK library selections - *E.coli* expressed CD59**	
TH2	TCPPCPAPELLGGPSVFLFPPKPKDTLMISRTPEVTCVVVDVSHEDPEVKFNWYVDGVEVH<br>NAKTKPREEQYNSTYRVVSVLTVLHQDWLNGKEYKCKVSNKALPAPIEKTISKAKGQPREP<br>QVYTLPPSRDELGSDYNYYNQVSLTCLVKGFYPSDIAVEWESNGQPENNYKTTPPVLDSDG<br>SFFLYSKLTVPQGRWYSGNVFSCSVMHEALHNHYTQKSLSLSPGK

TH3	TCPPCPAPELLGGPSVFLFPPKPKDTLMISRTPEVTCVVVDVSHEDPEVKFNWYVDGVEVH<br>NAKTKPREEQYNSTYRVVSVLTVLHQDWLNGKEYKCKVSNKALPAPIEKTISKAKGQPREP<br>QVYTLPPSRDELGTGYNYYNQVSLTCLVKGFYPSDIAVEWESNGQPENNYKTTPPVLDSDG<br>SFFLYSKLTVPQGRWYSGNVFSCSVMHEALHNHYTQKSLSLSPGK
TH5	TCPPCPAPELLGGPSVFLFPPKPKDTLMISRTPEVTCVVVDVSHEDPEVKFNWYVDGVEVH<br>NAKTKPREEQYNSTYRVVSVLTVLHQDWLNGKEYKCKVSNKALPAPIEKTISKAKGQPREP<br>QVYTLPPSRDELAWPLSYYNQVSLTCLVKGFYPSDIAVEWESNGQPENNYKTTPPVLDSDG<br>SFFLYSKLTVPQGRWYSGNVFSCSVMHEALHNHYTQKSLSLSPGK
TH6	TCPPCPAPELLGGPSVFLFPPKPKDTLMISRTPEVTCVVVDVSHEDPEVKFNWYVDGVEVH<br>NAKTKPREEQYNSTYRVVSVLTVLHQDWLNGKEYKCKVSNKALPAPIEKTISKAKGQPREP<br>QVYTLPPSRDELAWPLQYYNQVSLTCLVKGFYPSDIAVEWESNGQPENNYKTTPPVLDSDG<br>SFFLYSKLTVPQGRWYSGNVFSCSVMHEALHNHYTQKSLSLSPGK
TH7	TCPPCPAPELLGGPSVFLFPPKPKDTLMISRTPEVTCVVVDVSHEDPEVKFNWYVDGVEVH<br>NAKTKPREEQYNSTYRVVSVLTVLHQDWLNGKEYKCKVSNKALPAPIEKTISKAKGQPREP<br>QVYTLPPSRDELGSGYKYYNQVSLTCLVKGFYPSDIAVEWESNGQPENNYKTTPPVLDSDG<br>SFFLYSKLTVPQGRWYSGNVFSCSVMHEALHNHYTQKSLSLSPGK
**BER1x3_5NNK library selections - HEK293-6E -expressed CD59**	
BER5-1-1	TCPPCPAPELLGGPSVFLFPPKPKDTLMISRTPEVTCVVVDVSHEDPEVKFNWYVDGVEVH<br>NAKTKPREEQYNSTYRVVSVLTVLHQDWLNGKEYKCKVSNKALPAPIEKTISKAKGQPREP<br>QVYTLPPSRDELLWPLQYYNQVSLTCLVKGFYPSDIAVEWESNGQPENNYKTTPPVLDSDG<br>SFFLYSKLTVPQGRWYSGNVFSCSVMHEALHNHYTQKSLSLSPGK
BER5-1-3	TCPPCPAPELLGGPSVFLFPPKPKDTLMISRTPEVTCVVVDVSHEDPEVKFNWYVDGVEVH<br>NAKTKPREEQYNSTYRVVSVLTVLHQDWLNGKEYKCKVSNKALPAPIEKTISKAKGQPREP<br>QVYTLPPSRDELRWPLEYYNQVSLTCLVKGFYPSDIAVEWESNGQPENNYKTTPPVLDSDG<br>SFFLYSKLTVPQGRWYSGNVFSCSVMHEALHNHYTQKSLSLSPGK
BER5-1-4	TCPPCPAPELLGGPSVFLFPPKPKDTLMISRTPEVTCVVVDVSHEDPEVKFNWYVDGVEVH<br>NAKTKPREEQYNSTYRVVSVLTVLHQDWLNGKEYKCKVSNKALPAPIEKTISKAKGQPREP<br>QVYTLPPSRDELLWPLSYYNQVSLTCLVKGFYPSDIAVEWESNGQPENNYKTTPPVLDSDG<br>SFFLYSKLTVPQGRWYSGNVFSCSVMHEALHNHYTQKSLSLSPGK
BER5-2-5	TCPPCPAPELLGGPSVFLFPPKPKDTLMISRTPEVTCVVVDVSHEDPEVKFNWYVDGVEVH<br>NAKTKPREEQYNSTYRVVSVLTVLHQDWLNGKEYKCKVSNKALPAPIEKTISKAKGQPREP<br>QVYTLPPSRDELPSSGRYYNQVSLTCLVKGFYPSDIAVEWESNGQPENNYKTTPPVLDSDG<br>SFFLYSKLTVPQGRWYSGNVFSCSVMHEALHNHYTQKSLSLSPGK
BER5-2-7	TCPPCPAPELLGGPSVFLFPPKPKDTLMISRTPEVTCVVVDVSHEDPEVKFNWYVDGVEVH<br>NAKTKPREEQYNSTYRVVSVLTVLHQDWLNGKEYKCKVSNKALPAPIEKTISKAKGQPREP<br>QVYTLPPSRDELTWPLKYYNQVSLTCLVKGFYPSDIAVEWESNGQPENNYKTTPPVLDSDG<br>SFFLYSKLTVPQGRWYSGNVFSCSVMHEALHNHYTQKSLSLSPGK
BER5-1-9	TCPPCPAPELLGGPSVFLFPPKPKDTLMISRTPEVTCVVVDVSHEDPEVKFNWYVDGVEVH<br>NAKTKPREEQYNSTYRVVSVLTVLHQDWLNGKEYKCKVSNKALPAPIEKTISKAKGQPREP<br>QVYTLPPSRDELGSSGRYYNQVSLTCLVKGFYPSDIAVEWESNGQPENNYKTTPPVLDSDG<br>SFFLYSKLTVPQGRWYSGNVFSCSVMHEALHNHYTQKSLSLSPGK
BER5-1-11	TCPPCPAPELLGGPSVFLFPPKPKDTLMISRTPEVTCVVVDVSHEDPEVKFNWYVDGVEVH<br>NAKTKPREEQYNSTYRVVSVLTVLHQDWLNGKEYKCKVSNKALPAPIEKTISKAKGQPREP<br>QVYTLPPSRDELPQSGRYYNQVSLTCLVKGFYPSDIAVEWESNGQPENNYKTTPPVLDSDG<br>SFFLYSKLTVPQGRWYSGNVFSCSVMHEALHNHYTQKSLSLSPGK
BER5-1-14	TCPPCPAPELLGGPSVFLFPPKPKDTLMISRTPEVTCVVVDVSHEDPEVKFNWYVDGVEVH<br>NAKTKPREEQYNSTYRVVSVLTVLHQDWLNGKEYKCKVSNKALPAPIEKTISKAKGQPREP<br>QVYTLPPSRDELKWPLQYYNQVSLTCLVKGFYPSDIAVEWESNGQPENNYKTTPPVLDSDG<br>SFFLYSKLTVPQGRWYSGNVFSCSVMHEALHNHYTQKSLSLSPGK
BER5-1-15	TCPPCPAPELLGGPSVFLFPPKPKDTLMISRTPEVTCVVVDVSHEDPEVKFNWYVDGVEVH<br>NAKTKPREEQYNSTYRVVSVLTVLHQDWLNGKEYKCKVSNKALPAPIEKTISKAKGQPREP<br>QVYTLPPSRDELGYTLRYYNQVSLTCLVKGFYPSDIAVEWESNGQPENNYKTTPPVLDSDG<br>SFFLYSKLTVPQGRWYSGNVFSCSVMHEALHNHYTQKSLSLSPGK
BER5-1-16	TCPPCPAPELLGGPSVFLFPPKPKDTLMISRTPEVTCVVVDVSHEDPEVKFNWYVDGVEVH<br>NAKTKPREEQYNSTYRVVSVLTVLHQDWLNGKEYKCKVSNKALPAPIEKTISKAKGQPREP

	QVYTLPPSRDELPYSGRYYNQVSLTCLVKGFYPSDIAVEWESNGQPENNYKTTPPVLDSDG SFFLYSKLTVPQGRWYSGNVFSCSVMHEALHNHYTQKSLSLSPGK
**Antigens**	
CD59-*E.coli*	MGSSHHHHHHSSGLVPRGSHMLQCYNCPNPTADCKTAVNCSSDFDACLITKAGLQVYNKC WKFEHCNFNDVTTRLRENELTYYCCKKDLCNFNEQLEN
CD59-HEK	HHHHHHHHGSTGENLYFQGASLQCYNCPNPTADCKTAVNCSSDFDACLITKAGLQVYNKC WKFEHCNFNDVTTRLRENELTYYCCKKDLCNFNEQLEN
