# Supplementary material for: Bispecific mAb2 Antibodies Targeting CD59 Enhance the Complement-Dependent Cytotoxicity Mediated by Rituximab
Source: Int J Mol Sci. 2022 May 6;23(9):5208. doi: 10.3390/ijms23095208 (PMC9103234; doi:10.3390/ijms23095208)
Supplement: Supplementary file 1 [file ijms-23-05208-s001.zip › Table_S2.pdf]

Table S2. Kinetic parameters of binding of bispecific antibodies to CD59, determined with biolayer interferometry

mAb[2]	Antigen source	K_D (x10^{-9} M)	K_{on} (x10^5 1/Ms)	K_{dis} (x10^{-4} 1/s)
RX-BER1	*E. coli*	84.7	1.21	103
RX-BER2	*E. coli*	285	0.686	196
RX-BER3	*E. coli*	79.1	1.51	119
RX-BER1x3	*E. coli*	71.9	1.73	124
RX-BER5-1-3	*E. coli*	2.8	1.79	5.07
	HEK293-6E	40.4	2.44	98.4
