# Supplementary material for: Bispecific mAb2 Antibodies Targeting CD59 Enhance the Complement-Dependent Cytotoxicity Mediated by Rituximab
Source: Int J Mol Sci. 2022 May 6;23(9):5208. doi: 10.3390/ijms23095208 (PMC9103234; doi:10.3390/ijms23095208)
Supplement: Supplementary file 1 [file ijms-23-05208-s001.zip › Table_S3.pdf]

Supplementary Table S3. Nucleotide sequences of primers used for library construction and cloning.

Oligonucleotide	Sequence
**Antigen expression**	
Cloning *E. coli* CD59	
CD59nde1	acgtcatatgctgcagtgctacaattg
CD59bam2	acgcggatccttatcagttctccagctgctcgttg
Cloning HEK CD59	
CD59nhe1	acgtgctagcctgcagtgctacaattgtc
CD59bam2	acgcggatccttatcagttctccagctgctcgttg
**Yeast libraries**	
AB01-forward	gaaccacaggtgtacaccctgcccccatcccgggatgagnnknnknnknnknnkgtcagcctgacctgcctg gtcaaag
AB03-forward	gaaccacaggtgtacaccctgcccccatcccgggatgagctgnnknnknnknnknnknnknnkaaccaggt cagcctgacctgcctggtcaaag
EF01-reverse	gtggttgtgcagagcctcatgcatcacggagcatgagaamnnmnnmnnmnnmnnccacctmnnmnn mnncacggtgagcttgctgtagaggaagaag
EF03-reverse	gtggttgtgcagagcctcatgcatcacggagcatgagaagacgttcccmnnmnnccacctmnnmnnmnn cacggtgagcttgctgtagaggaa
Screening of the insert in pyd1dxdem vector	
pydfwd	agtaacgtttgtcagtaattgc
pydrev	gtcgattttgttacatctacac
Shuffling of Fcab clone sequences	
ABnest	ccaagggccagcctcgagaaccacaggtgtacac
3CD	ggaagaaggagccgtcggagtccagcac
5EF	gtgctggactccgacggctccttcttcc
upnest	gtgtgtagtggttgtgcagagcctcatgcatcacggag
Pool expansion libraries	
BER1x3_4NNK	gtacaccctgcccccatcccgggatgagctgnnsnnsnnsnnstactacaaccaggtcagcc
BER1x3_5NNK	gtacaccctgcccccatcccgggatgagctgnnsnnsnnsnnsnnstactacaaccaggtcagcc
**Cloning to mammalian expression vectors**	
PCR of mutated CH3 domain	
ABnest	ccaagggccagcctcgagaaccacaggtgtacac
CH3sbam	gattggatcctcatttacccggagacagg
Screening of the insert in pTT vector	
pTTfwd	gccatacacttgagtgacaatgacatc
pTTrev	ccaaatgatttgccctcccatatgtc
